# Pyrazole–Cyclotriphosphazene Hybrids: Synthesis, Structural Insights, and Cytotoxic Effects against Pancreatic Cancer Cells

**DOI:** 10.1021/acsomega.5c11955

**Published:** 2026-04-08

**Authors:** Ceylan Mutlu Balcı, Basak Dalbayrak, Esma Mutlu, Duygu Palabıyık, Elif Damla Arısan

**Affiliations:** † Department of Chemistry, Faculty of Basic Sciences, 52962 Gebze Technical University, Gebze 41400, Kocaeli, Turkey; ‡ Institute of Biotechnology, Gebze Technical University, Gebze 41400, Kocaeli, Turkey

## Abstract

Pancreatic cancer carries an extremely poor long-term survival, with under 5% of patients living more than five years after diagnosis. Thus, the demand for innovative therapeutic medications to treat pancreatic cancer is essential. In this study, inorganic–organic hybrid molecules were synthesized by performing nucleophilic substitution reactions of 4-chloro-2-(1*H*-pyrazol-3-yl)­phenol (**2a**) containing the pyrazole scaffold, which has a biologically active skeleton, with hexachlorocyclotriphosphazene, HCCTP (**1**). The reactions were carried out with two different bases (NaH and Et_3_N) to isolate various cyclotriphosphazene derivatives that could form due to the tautomerization of the pyrazole skeleton and to compare the reaction yields. Products were formed in which HCCTP (**1**) was substituted at the monospiro (**3a**), dispiro (*trans* **4a-I** and *cis* **4a-II**), and trispiro *(cis–trans–trans* **5a-I** and all-*cis* **5a-II**) positions of the chloro phenol pyrazole group. The structures of these newly synthesized hybrid molecules have been elucidated using various spectroscopic techniques (^31^P, ^1^H, ^13^C NMR, and mass spectroscopy), elemental analysis, and single-crystal X-ray diffraction techniques. For the purpose of evaluating biological properties (*in vitro* studies on potential pancreatic cancer cells), monospiro, *trans*- dispiro, and *cis–trans–trans* trispiro 2-(1*H*-pyrazol-3-yl)­phenol substituted cyclotriphosphazene compounds (**3b**, **4b-I**, and **5b-I**) were synthesized according to the literature. The biological properties of all compounds (**3a**, **4a-I**, **5a-I**, **3b**, **4b-I**, and **5b-I**) were investigated for the first time in pancreatic cancer cell lines (PANC-1 and MIA PaCa-2). Compound **3a** displays potent anticancer activity against PANC-1 (IC_50_ = 24.57 μg/mL) and MIA PaCa-2 (IC_50_ = 23.30 μg/mL) cells.

## Graphical abstract

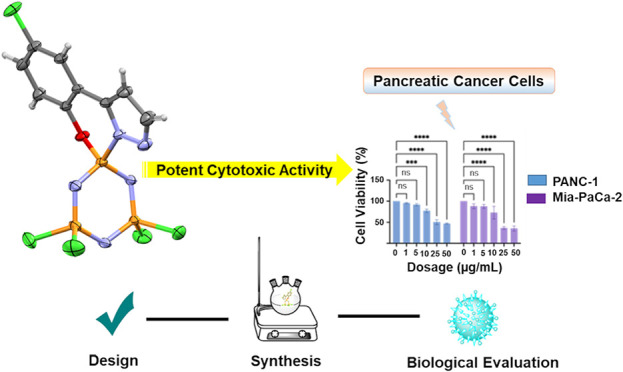

## 1 Introduction

Cancer is a complex disease that kills millions of people and is caused by unchecked cell proliferation. Globally, 18.1 million people received a cancer diagnosis in 2018, and by 2040, that is predicted to rise to 29.4 million.^1^ According to reports, pancreatic cancer is the leading cause of cancer-related deaths worldwide and is one of the deadliest tumor kinds.^2,3^ Thus, new therapeutic medications are desperately needed to treat pancreatic cancer.

Heterocyclic structures have garnered significant interest due to their presence in many biologically active molecules.^4–6^ It is well established that nitrogen-containing heterocycles represent privileged scaffolds in drug discovery, being found in numerous pharmacophores that exhibit anticancer, anti-inflammatory, antimicrobial, and antiviral properties.^7–12^ Pyrazole derivatives, in particular, are notable for their five-membered ring structure, which enables them to interact with diverse biological targets through hydrogen bonding and hydrophobic interactions.^12–15^ This structural versatility has led to their inclusion in several clinically approved drugs, with research reporting vigorous antibacterial, antifungal, and antitumor activity for pyrazole-based compounds.^12–15^ Similarly, cyclotriphosphazene-based molecules, due to the tunable nature of their phosphorus–nitrogen backbone, allow for the introduction of a variety of organic groups, resulting in stable and versatile inorganic–organic hybrid systems.^16–20^ Recent study has shown that dispiro and trispiro (*N*/*N*) cyclotriphosphazenes exhibit pronounced conformational diversity and chirality arising from spiro-fused ring systems and these structural attributes have been correlated with electrochemical behavior and optoelectronic performance, including applications in dye-sensitized solar cells.^17^ In the study reported by Cemaloğlu et al.,^18^ the cyclotriphosphazene core was employed as a versatile molecular platform for the design and synthesis of hybrid compounds bearing unsymmetrical pendant arms. Structure–activity relationship (SAR) investigations revealed that both the nature and the spatial distribution of the heterocyclic substituents on the cyclotriphosphazene ring significantly influenced antimicrobial and cytotoxic properties.^18^ Moreover, direct functionalization of the cyclotriphosphazene scaffold with antioxidant and chromophore groups, such as butylated oxyanisole and hydroxyflavone units, has significantly broadened the application scope of phosphazene-based compounds toward biological and therapeutic research.^19,20^ Sönmez et al.^19^ reported the synthesis of cyclotriphosphazene derivatives incorporating butylated oxyanisole moieties, where biological evaluations revealed that the cyclotriphosphazene scaffold plays an active role in enhancing antioxidant activity. Similarly, Kitmür et al.^20^ designed cyclotriphosphazene derivatives bearing hydroxyflavone groups, utilizing the substitutional versatility of the phosphazene ring to achieve multifunctional architectures with pronounced biological efficacy. The resulting compounds exhibited notable antioxidant properties and significant cytotoxic effects against breast cancer cell lines. In this context, the introduction of aromatic, heterocyclic, and amine functionalities onto the cyclotriphosphazene core has been shown to enhance cytotoxic and antimicrobial activities against various cancer cell lines and bacterial strains.^18–21^ Also, hybrid systems combining pyrazole and phosphazene hold considerable promise for therapeutic development due to the convergence of their pharmacological advantages, including not only enhanced biological activity but also improved chemical stability and functionalization potential.^16,22,23^ Thus, the rational design and biological evaluation of these new cyclotriphosphazene-pyrazole hybrids remain a compelling and evolving frontier in medicinal chemistry.

In this study, the reactions of 4-chloro-2-(1*H*-pyrazol-3-yl)­phenol (**2a**) with HCCTP (**1**) were first carried out in the presence of Et_3_N base at different mole ratios. As a result of these reactions, it was observed that spiro structures were formed, with bonding occurring through the *N* ^2^ atom of the pyrazole scaffold. To compare product diversity and yield, the reactions were repeated in the presence of NaH, a stronger base. Spiro derivatives **3a**, **4a-I**, and **5a-I** were isolated (Scheme 1). The structures of the obtained chloro phenol pyrazole substituted products (**3a**, **4a-I** and **5a-I**) were characterized by NMR (^31^P, ^1^H, ^13^C) spectroscopy, mass, and elemental analyses. The structures of all compounds were also confirmed using the single-crystal X-ray diffraction technique. In addition, spiro phenol pyrazole cyclotriphosphazene compounds from the literature {products obtained in the reaction of 2-(1*H*-pyrazol-3-yl)­phenol (**2b)** with HCCTP (**1**)} were resynthesized in the study (**3b**, **4b-I**, and **5b-I**) (Table 1) to examine their biological properties using the MTT and colony assays with fluorescence imaging on PANC-1 and MIA PaCa-2 cell lines and determine whether the chlorine atom would make any difference in the structures. Additionally, all compounds (**3a**, **3b**, **4a-I**, **4b-I** and **5b-I**) were examined for their antimicrobial activities against Gram-positive (*S. aureus*) and Gram-negative (*E. coli*, *P. aeruginosa*) bacteria. At the same time, the thermal properties of all compounds (**3a**, **3b**, **4a-I**, **4b-I**, **5a-I** and **5b-I**) were determined by thermal gravimetric analysis (TGA).

**Scheme 1:**
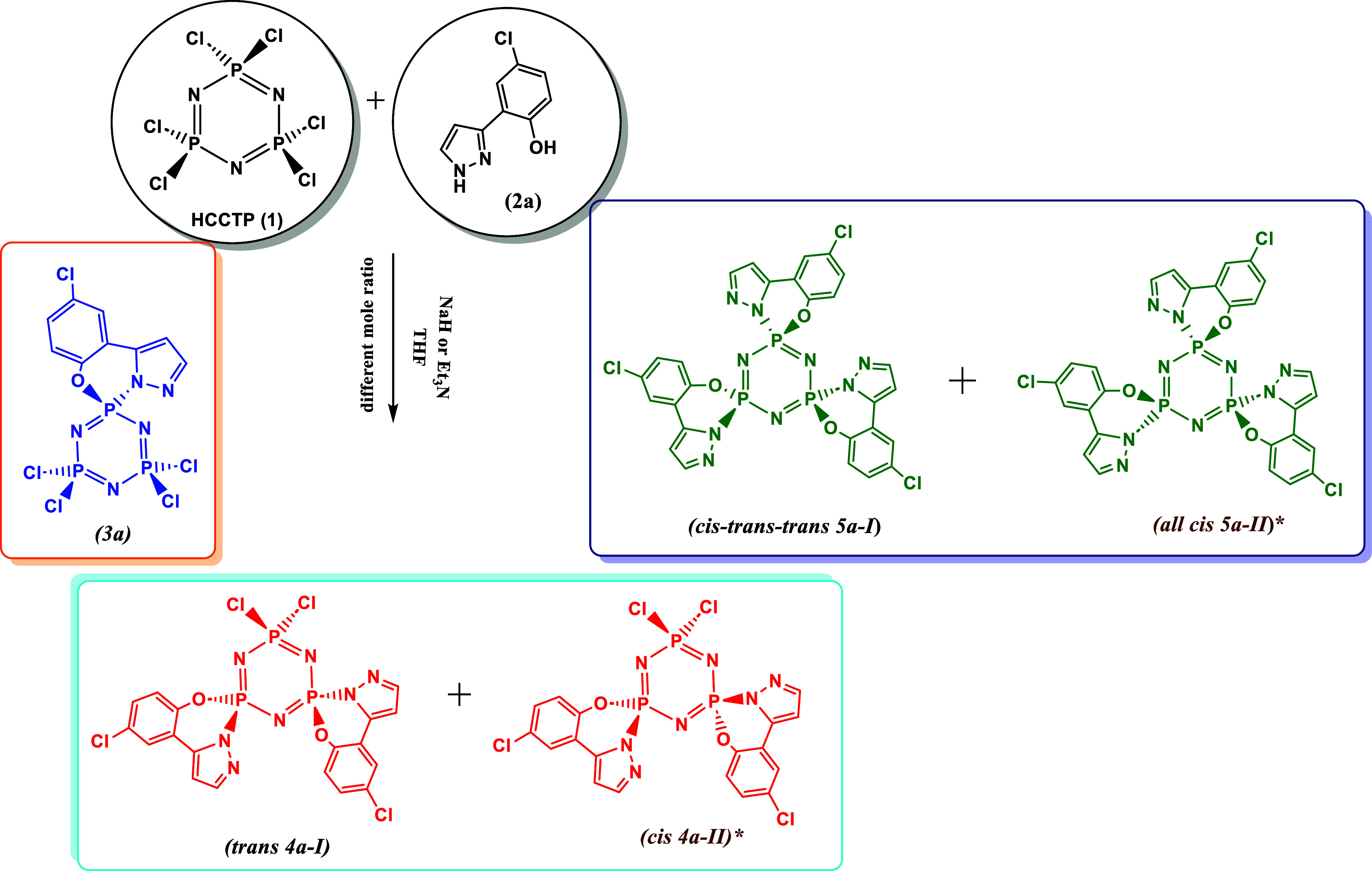
Products of the Reaction of Compounds HCCTP (**1**) with the 4-chloro-2-(1*H*-pyrazole-3-yl)­phenol (**2a**)

**Table 1:**
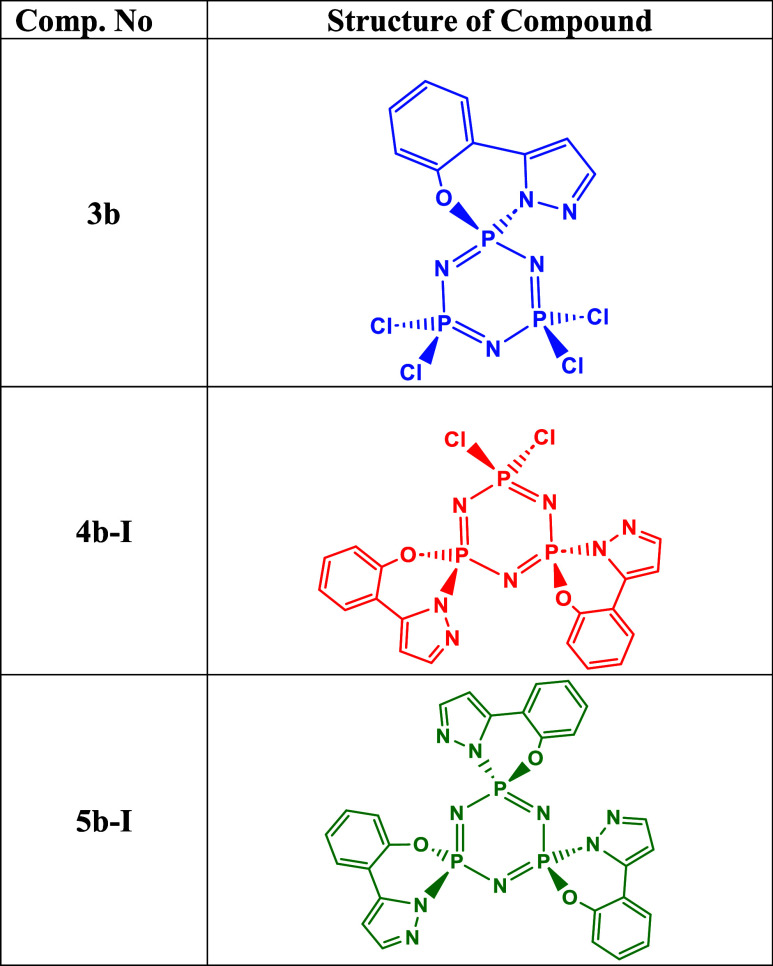
Re-Synthesized Compounds (**3b, 4b-I** and **5b-I**) for Biological Investigations

## 2 Experimental Section

### 2.1 Materials and Physical Measurements

Hexachlorocyclotriphosphazene, HCCTP (Sigma-Aldrich, 99%) was purified by fractional crystallization from *n*-hexane (Merck, ≥98). 2-(1*H*-pyrazol-3-yl)­phenol (Sigma-Aldrich, 97%), 4-chloro-2-(1*H*-pyrazol-3-yl)­phenol (Sigma-Aldrich, 98%), dichloromethane, DCM (Merck, ≥99.99), *n*-hexane (Merck, ≥98), *n*-heptane (Merck, ≥99) and the chiral solvating agent (R)-(+)-2,2,2-trifluoro-1-(9-anthryl)­ethanol (CSA) (Sigma, ≥98) were used commercially. Tetrahydrofuran, THF (Sigma-Aldrich, ≥99.9), was distilled over Na–K alloy in an argon atmosphere. Sodium hydride, 60% dispersion in mineral oil (Merck); before use, the oil was removed by washing with *n*-hexane followed by decantation. The reactions were performed under an argon atmosphere. Deuterated chloroform (CDCl_3_, 99.8 atom % D) for NMR spectroscopy was also obtained from Merck. Analytical Thin Layer Chromatography (TLC) was performed on Merck silica gel plates (Merck, Kieselgel 60, 0.25 mm thickness) with F_254_ indicator. Column chromatography was performed on silica gel (Merck, Kieselgel 60, 230–400 mesh; for 3 g crude mixture, 100g silica gel was used). Elemental analysis data were obtained using an Elementar Vario MICRO Cube. Molecular masses were measured using a Bruker MALDI–TOF (Matrix-Assisted Laser Desorption/Ionization-Time-Of-Flight) spectrometer using 2,5-dihydroxybenzoic acid (Alfa Aesar, 99%) as a matrix for compounds **5a-I**, and 1,8,9-trihydroxyanthracene (TCI, > 95%) as a matrix for compounds **3a** and **4a-I**. ^31^P NMR spectra were recorded for all compounds in CDCl_3_ on a Varian INOVA 500 MHz spectrometer using 85% H_3_PO_4_ as an external reference for ^31^P NMR measurements. Thermal analyses were performed on Mettler Toledo TGA/SDTA 851.

### 2.2 X-ray Data Collection and Structure Refinement

The slow solvent evaporation procedure yielded 4-chloro-2-(1*H*-pyrazol-3-yl)­phenol appended spiro cyclotriphosphazene (**3a**, **4a-I**, and **5a-I**) crystals which are suitable for single-crystal X-ray analysis studies. In a solvent system consisting of dichloromethane and *n*-heptane (1:3), single crystals were formed at room temperature. Under a polarized microscope, suitable single crystals of compounds **3a**, **4a-I**, and **5a-I** were determined and oil containing perfluoropolyether was used to clean them. The crystal structures of the compounds **3a**, **4a-I**, and **5a-I** were then verified using single-crystal X-ray crystallography. Intensity data were recorded on a Bruker APEX II QUAZAR diffractometer using monochromatized Mo–K_α_ x-radiation (λ = 0.71073 Å). Absorption correction was performed by the multiscan method implemented in SADABS,^24^ and space groups were determined in APEX2.^25^ The structures were solved using SHELXT^26^ and then refined by full-matrix least-squares refinements on F^2^ using SHELXL^27^ in the Olex2 program package.^28^ All non-hydrogen atoms were refined anisotropically using all reflections with *I* > 2σ­(*I*). The final geometrical calculations were carried out with PLATON^29^ and MERCURY^30^ programs, and the molecular drawings were done with DIAMOND^31^ program. Structure determination has been deposited with the Cambridge Crystallographic Data Centre with reference codes as 2495906 for compound **3a**, 2495907 for compound **4a-I**, and 2495908 for compound **5a-I**.

### 2.3 Syntheses

Compounds **3b**,^16^ **4b-I**,^16,23^ and **5b-I** ^23^ were synthesized and isolated in pure form as described in the literature for the study of their biological properties.

#### 2.3.1 Reaction of 4-chloro-2-(1*H*-pyrazol-3-yl)­phenol (2a) with HCCTP (1) in a 1:1.2 mol Ratio

The compound **1** (0.60 g, 1.73 mmol) was dissolved by using dry THF (10 mL) in a beaker and transferred to a 100 mL three-neck round-bottom flask. Then, under an inert argon (Ar) atmosphere, Et_3_N (0.29 mL, 4.16 mmol) was added to the reaction medium with the aid of a pipet. After this step, 4-chloro-2-(1*H*-pyrazol-3-yl)­phenol (**2a**) (0.40 g, 2.08 mmol) in dry THF (10 mL) was dropped into the stirred solution. The reaction mixture was stirred for a further 24 h at ambient temperature. The reaction progress was monitored using thin-layer chromatography (TLC) with *n*-hexane-dichloromethane (2:1) solvent system. Except for the starting compound, HCCTP (**1**), it was seen that one new product was formed. To separate the insoluble salts and other residues, the reaction was filtered using a G4 filter. After that, high vacuum was applied to remove the solvent. Column chromatography was used to purify the crude reaction mixture, with *n*-hexane-dichloromethane (2:1) serving as the eluent. First, the HCCTP (**1**) (0.09 g, 14%) was isolated from the column, and then the monospiro compound **3a** was eluted from the column. The dichloromethane-*n-*heptane (1:3) solvent mixture was used to crystallize compound **3a**, yielding colorless single crystals.

Anal. Calc. for **3a**; C_9_H_5_Cl_5_N_5_OP_3_: C, 23.03; H, 1.07; N, 14.92%, M, 469.34.

Compound **3a** (0.62 g, 77%, mp 234 °C), Found: C, 23.11; H, 1.13; N, 14.81%, [M]^+^, 469.01 *m*/*z*. ^31^P {^1^H} NMR decoupled, CDCl_3_, 298 K, AX_2_ spin system, δ (ppm); 24.77 2x­[PCl_2_] and −7.30 [P­(NO-spiro)], ^2^J_AX_ = 69.6 Hz.

^1^H NMR, CDCl_3_, 298 K, δ (ppm); 7.94 (m, 1*H*_9_), 7.60 (d, 1*H*_5_, *J* = 2.5 Hz), 7.30 (dt, 1*H*_3_, *J* = 8.8 Hz, 2.5 Hz), 7.19 (d, 1*H*_2_, *J* = 8.8 Hz), 6.70 (m, 1*H*_8_).

^13^ C NMR, CDCl_3_, 298 K, δ (ppm); C_1_; 145.85 (d, *J* = 18.4 Hz), C_9_; 145.28 (d, *J* = 8.4 Hz), C_7_; 140.98 (d, *J* = 10.2 Hz), C_4_; 134.76 (s), C_5_; 129.61 (s), C_2_; 124.87 (s), C_3_; 119.66 (d, *J* = 5.1 Hz), C_6_; 115.45 (s), C_8_; 102.77 (d, *J* = 5.4 Hz).

#### 2.3.2 Reaction of 4-chloro-2-(1*H*-pyrazol-3-yl)­phenol (2a) with HCCTP (1) in a 1:2.2 mol Ratio

HCCTP (**1)** (0.60 g, 1.73 mmol) was dissolved by using dry THF (20 mL) in a beaker and transferred to a 100 mL three-neck round-bottom flask. Then, under an inert (Ar) atmosphere, Et_3_N (1.10 mL, 7.62 mmol) was added to the reaction medium with the aid of a pipet. After this step, 4-chloro-2-(1*H*-pyrazol-3-yl)­phenol (**2a**) (0.74 g, 3.81 mmol) in dry THF (15 mL) was dropped into the stirred solution. The reaction mixture was stirred for a further 24 h at ambient temperature. The reaction progress was monitored using thin-layer chromatography (TLC) with *n*-hexane-tetrahydrofuran (2:1) solvent system. Except for the monospiro compound **3a**, three different spots were observed on the TLC sheet. To separate the insoluble salts and other residues, the reaction was filtered using a G4 filter. After that, high vacuum was applied to remove the solvent. Column chromatography was used to purify the crude reaction mixture, with *n*-hexane-tetrahydrofuran (2:1) serving as the eluent. First, the monospiro (**3a**) (4 mg, 4%) was isolated from the column; then, the *trans*-dispiro (**4a-I**) was eluted from the column. Finally, the trispiro *cis–trans–trans* compound (**5a-I**) was isolated. Another spot on the TLC sheet could not be isolated despite more polar solvent systems being also tried for column. The dichloromethane-*n-*heptane (1:3) solvent mixture was used to crystallize compound **4a-I**, yielding colorless single crystals.

Anal. Calc. for **4a-I**; C_18_H_10_Cl_4_N_7_O_2_P_3_: C, 36.58; H, 1.71; N, 16.59%, M, 591.1.

Compound **4a-I** (0.57 g, 56%, mp 218 °C), Found: C, 36.66; H, 1.80; N, 16.52%, [M]^+^, 591.2 *m*/*z*. ^31^P {^1^H} NMR decoupled, CDCl_3_, 298 K, A_2_X spin system, δ (ppm); 28.65 [PCl_2_] and −2.21 2x­[P­(NO-spiro)], ^2^J_AX_ = 77.4 Hz (Figure S1).

^1^H NMR, CDCl_3_, 298 K, δ (ppm); 7.96 (d, 2*H*_9_, *J* = 1.8 Hz), 7.58 (d, 2*H*_5_, *J* = 2.4 Hz), 7.26 (dd, 2*H*_3_, *J* = 8.9 Hz, 2.4 Hz), 7.22 (d, 2*H*_2_, *J* = 8.8 Hz), 6.71 (d, 2*H*_8_, *J* = 1.8 Hz) (Figure S2).

^13^ C NMR, CDCl_3_, 298 K, δ (ppm); C_1_; 145.64 (t, *J* = 9.3 Hz), C_9_; 145.43 (t, *J* = 8.1 Hz), C_7_; 141.15 (s), C_4_; 134.95 (s), C_5_; 129.77 (s), 129.53 (s), C_2_; 123.78 (s), C_3_; 119.88 (d, *J* = 5.4 Hz), C_6_; 115.58 (s), C_8_; 102.66 (s) (Figure S3).

Anal. Calc. for **5a-I**; C_27_H_15_Cl_3_N_9_O_3_P_3_: C, 45.50; H, 2.12; N, 17.69%, M, 712.8.

Compound **5a-I** (0.03 g, 2%, mp 260 °C), Found: C, 45.57; H, 2.20; N, 17.60%, [M+2H]^+^, 714.4 *m*/*z*. ^31^P {^1^H} NMR decoupled, CDCl_3_, 298 K, AB_2_ spin system, δ (ppm); 1.96 3x [P­(NO-spiro)] (Figure S4).

^1^H NMR, CDCl_3_, 298 K, δ (ppm); 8.00 (br, 3*H*_9_), 7.52 (m, 3*H*_5_), 7.30 (d, 1*H*_3_, ^*3*^J_HH_ = 8.9 Hz), 7.24­(m, 2*H*_3_ and 3*H*_2_), 6.67 (br, 1*H*_8_), 6.64 (br, 2*H*_8_) (Figure S5).

^13^ C NMR, CDCl_3_, 298 K, δ (ppm); C_1_; 146.60 (m), C_9_; 146.42 (m), C_7_; 141.08 (m), C_4_; 134.88 (s), C_5_; 129.48 (s), 129, 31 (s), C_2_; 123.63 (d, ^3^J_PC_= 8.1 Hz), C_3_, 119.99 (m), C_6_; 115.75 (s), C_8_; 102.46 (m) (Figure S6).

#### 2.3.3 Reaction of 4-chloro-2-(1*H*-pyrazol-3-yl)­phenol (2a) with HCCTP (1) in a 1:3.2 mol Ratio

HCCTP (**1**) (0.40 g, 1.15 mmol) was dissolved by using dry THF (10 mL) in a beaker and transferred to a 50 mL three-neck round-bottom flask. Then, under an inert (Ar) atmosphere, Et_3_N (1.03 mL, 7.36 mmol) was added to the reaction medium with the help of a pipet. After this step, 4-chloro-2-(1*H*-pyrazol-3-yl)­phenol (**2a**) (0.72 g, 3.68 mmol) in dry THF (10 mL) was dropped into the stirred solution. The reaction mixture was stirred for a further 24 h at ambient temperature. The reaction progress was monitored using thin-layer chromatography (TLC) with *n*-hexane-tetrahydrofuran (2:1) solvent system. Two products (**4a-I** and **5a-I**) were observed on the TLC sheet. To separate the insoluble salts and other residues, the reaction was filtered using a G4 filter. After that, high vacuum was applied to remove the solvent. Column chromatography was used to purify the crude reaction mixture, with *n*-hexane-tetrahydrofuran (2:1) serving as the eluent. First, the *trans* dispiro (**4a-I**) (0.34 g, 50%) was isolated from the column. Then, the trispiro *cis–trans–trans* compound (**5a-I**) (0.13 g, 16%) was isolated. The dichloromethane-*n-*heptane (1:3) solvent mixture was used to crystallize compound **5a-I**, yielding colorless single crystals.

#### 2.3.4 General Procedure Used for Reaction of HCCTP (1) with 4-chloro-2-(1*H*-pyrazol-3-yl)­phenol (2a) in the Presence of NaH

In a 50 mL three-necked round-bottom flask, HCCTP (**1**) was dissolved by using 10 mL of dry THF. In the stirred solution, 4-chloro-2-(1*H*-pyrazol-3-yl)­phenol (**2a**) was added (in 10 mL of dry THF) under an Ar atmosphere. After 5 mL of dry THF was rapidly mixed with NaH (60% oil suspension). To remove sodium chloride and any other insoluble residues, the reaction was filtered after being agitated for an additional 24 h at room temperature. TLC sheets were used to monitor the reaction. Column chromatography was used to separate the products from the mixed reaction mixture after the solvent was removed under high vacuum. Reactions were carried out at mole ratios of 1:1.2, 1:2.2, and 1:3.2 by using the same reaction procedure. The column solvent system, g and mmol amounts for these reactions, and isolated product yields of the compounds were given in Table 2.

**Table 2:** Preparation of the Products Using NaH Base

HCCTP (**1**)		4-chloro-2-(1*H*-pyrazol-3-yl)phenol (**2a**)		NaH			
g	mmol	g	mmol	g	mmol	Chromatography mobile phase (*n*-hexane:THF)	Isolated product yield (Cpd.; % yield)
0.60	1.73	0.40	2.08	0.17	4.16	3:1	**3a;** 74
							**4a-I;** 12
0.16	0.46	0.20	1.01	0.08	2.02	2:1	**3a;** 10
							**4a-I;** 59
							**5a-I;** 3
0.10	0.29	0.18	0.93	0.07	1.86	2:1	**5a-I;** 85

### 2.4 Cellular Experiments

#### 2.4.1 Cell Viability Assay

PANC-1 (CRL-1469) and MIA PaCa-2 (CRL-1420) cells were purchased from American Type Culture Collection (ATCC). PANC-1 and MIA PaCa-2 cells were grown in Dulbecco’s Modified Eagle Medium (DMEM) (Gibco) with the addition of 10% fetal bovine serum (FBS) (PAN Biotech) and 1% penicillin/streptomycin (PAN Biotech) at 37 °C in a humidified 5% CO2 incubator (NUVE, Turkiye). The cellular experiments were conducted under sterile conditions using a Class II Laminar Flow Cabinet (Grafen). All synthesized compounds were dissolved in DMSO (stock concentration of at least 3 mg/mL).

Cells were seeded at a density of 5 × 10^3^ per well in 96-well plates and allowed to adhere. After attachment, cells were treated with increasing concentrations of the compounds (1, 5, 10, 25, and 50 μg/mL) for 24 h. Following treatment, 10 μL of MTT reagent (3-(4,5-dimethylthiazol-2-yl)-2,5-diphenyl tetrazolium bromide, 5 mg/mL in PBS; NeoFroxx) was added to each well, and the plates were incubated at 37 °C for 4 h to allow formazan crystal formation. The formazan crystals were then dissolved in 100 μL of DMSO per well. The absorbance of the solubilized formazan was measured at 570 nm using a spectrophotometer (Bio-Tek, USA), which indicated cell viability in response to compound exposure.

Cells were seeded at a density of 2.5 × 10^3^ per well in 6-well plates and treated for 24 h with compounds at the same concentrations used in the MTT assay to assess their colonization potential. After treatment, the medium was replaced with fresh DMEM, and the cells were incubated for approximately 10 days to allow colony formation. Subsequently, cells were washed with 1X PBS, fixed with methanol: acetic acid (3:1) for 15 min, stained with 0.5% crystal violet in methanol for 15 min, washed with distilled water, and then imaged for analysis.

#### 2.4.2 Fluorescence Staining

PANC-1 and MIA PaCa-2 cells were seeded in 24-well plates at a density of 3 × 10^4^ per well and treated with optimized concentrations of compounds by MTT and colony formation assays for 24 h. After treatment, cells were washed once with 1x PBS and sequentially stained with several fluorescent probes: 10 μM H2-DCFDA (Ex/Em: 495/529 nm) to detect reactive oxygen species, 50 nM MitoSpy CMXRos (Biolegend) for mitochondrial labeling, 5 μg/mL DAPI (Ex/Em: 350/464 nm) for nuclear staining. Stained cells were then examined by fluorescence microscopy (SOIF), and the results from treated samples were compared to those from untreated controls to assess changes in ROS production, mitochondrial function, nuclear morphology, and cell viability.

The antimicrobial activities of the synthesized compounds were evaluated using the disk diffusion and minimum inhibitory concentration (MIC) methods against representative microorganisms, including the Gram-negative *Pseudomonas aeruginosa* and *Escherichia coli* and the Gram-positive *S. aureus.* A detailed experimental procedure is provided in the Supporting Information.

## 3 Results and Discussion

### 3.1 Structural Characterization of Isolated Products

In our previous study, it was observed that the pyrazole group could bind to the cyclotriphosphazene ring through two different bonding modes (*N* ^1^ and *N* ^2^), leading to distinct products *via* spiro and open structural arrangements.^16^ Therefore, in the present study, the reactions of HCCTP (**1**) with 4-Chloro-2-(1*H*-pyrazol-3-yl)­phenol (**2a**) were examined in detail in the presence of weak (Et_3_N) and strong (NaH) bases, with the aim of thoroughly investigating the potential product diversity and product yield. Chloro phenol pyrazol substituted spiro cyclotriphosphazene derivatives (**3a**, **4a-I**, **4a-II**, **5a-I** and **5a-II**) were formed (Scheme 1) and isolated products (**3a**, **4a-I** and **5a-I**) were characterized by using elemental analysis, MALDI-TOF mass spectrometry, ^1^H, ^13^C and ^31^P {^1^H} NMR spectroscopies. The analytical data including the elemental analyses, mass spectrometric results, and ^1^H, ^31^P {^1^H}, and ^13^C NMR data of isolated derivatives were given in the experimental part. ^31^P {^1^H} NMR spectroscopy was used to examine possible product formation in the reaction mixtures of compound **1** with **2a** using NaH and Et_3_N bases (Figures 1, 2, 3). Figure 4 provides an example of compound **3a**’s ^31^P {^1^H}, ^1^H, and ^13^C NMR spectra. The ^31^P {^1^H} NMR spectra of compound **4a-I**, which contains two chiral centers, with and without CSA, were acquired (Figure 5). Single-crystal X-ray diffraction was used to confirm the crystal structures of compounds **3a**, **4a-I**, and **5a-I** (Figures 6, 7, 8). The ^31^P {^1^H} NMR spectrum of the reaction of HCCTP (**1**) with **2a** in the presence of NaH showed the formation of a major (ca. 78%) product with an AX_2_ spin system (Figure 1a). Additionally, the presence of products with two A_2_X spin systems (*ca*. 18% and *ca*. 2%) containing two triplet and two doublet peak groups were determined, indicating that they may be configurational isomers of each other (Figure 1a). The MALDI-TOF mass analysis result of the main product (*ca*. 78%) isolated after purification by column chromatography was consistent with the mass value of the structure in which **2a** is monospiro bound to HCCTP (**1**). Indeed, the ^31^P {^1^H} NMR spectrum revealed a triplet resonance for the P­[NO-spiro] group at δ = −7.30 ppm and a doublet for the two PCl_2_ groups at δ = 24.77 ppm, indicative of an AX_2_ spin system. In the structure of compound **3a**, determined by single-crystal X-ray diffraction, it was clearly seen that the chloro phenol pyrazole group is attached to the structure as monospiro (Figure 6). The other isolated compound displayed an A_2_X spin system, with the PCl_2_ group observed as a triplet at δ = 28.65 ppm and the [P­(NO-spiro)] groups resonating at δ = −2.21 ppm, split into two signals. The integral ratios of the corresponding peaks 1:2 (PCl_2_: P­[NO-spiro]) further indicated that two chloro phenol pyrazole groups are spiro-bonded (Figure 1a). The mass spectrometric analysis (Figure S7) supported this structural assignment. Moreover, X-ray crystallographic data confirmed that the *N* and *O* atoms are positioned *trans* to each other, verifying that the structure is the *trans* dispiro cyclotriphosphazene compound (**4a-I**) (Figure 7).

**Figure 1:**
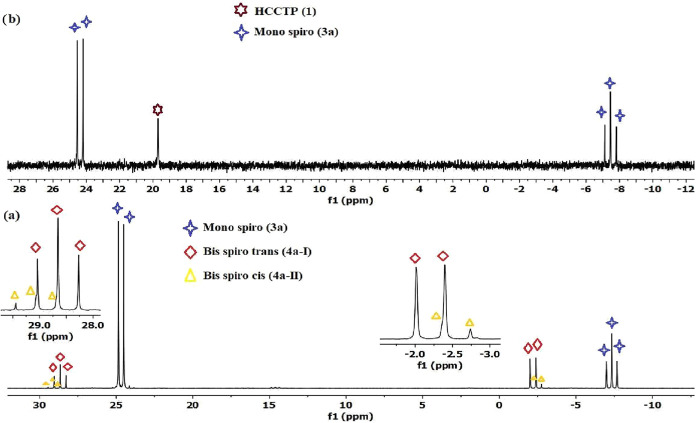
Proton-decoupled ^31^P­{^1^H} NMR spectrum of the reaction mixture of compound **1** with **2a** in a 1:1.2 mol ratio in THF solution with (a) NaH base (b) Et_3_N base; the reaction mixture was filtered, and the solvent removed prior to dissolving in CDCl_3_ solution.

**Figure 2:**
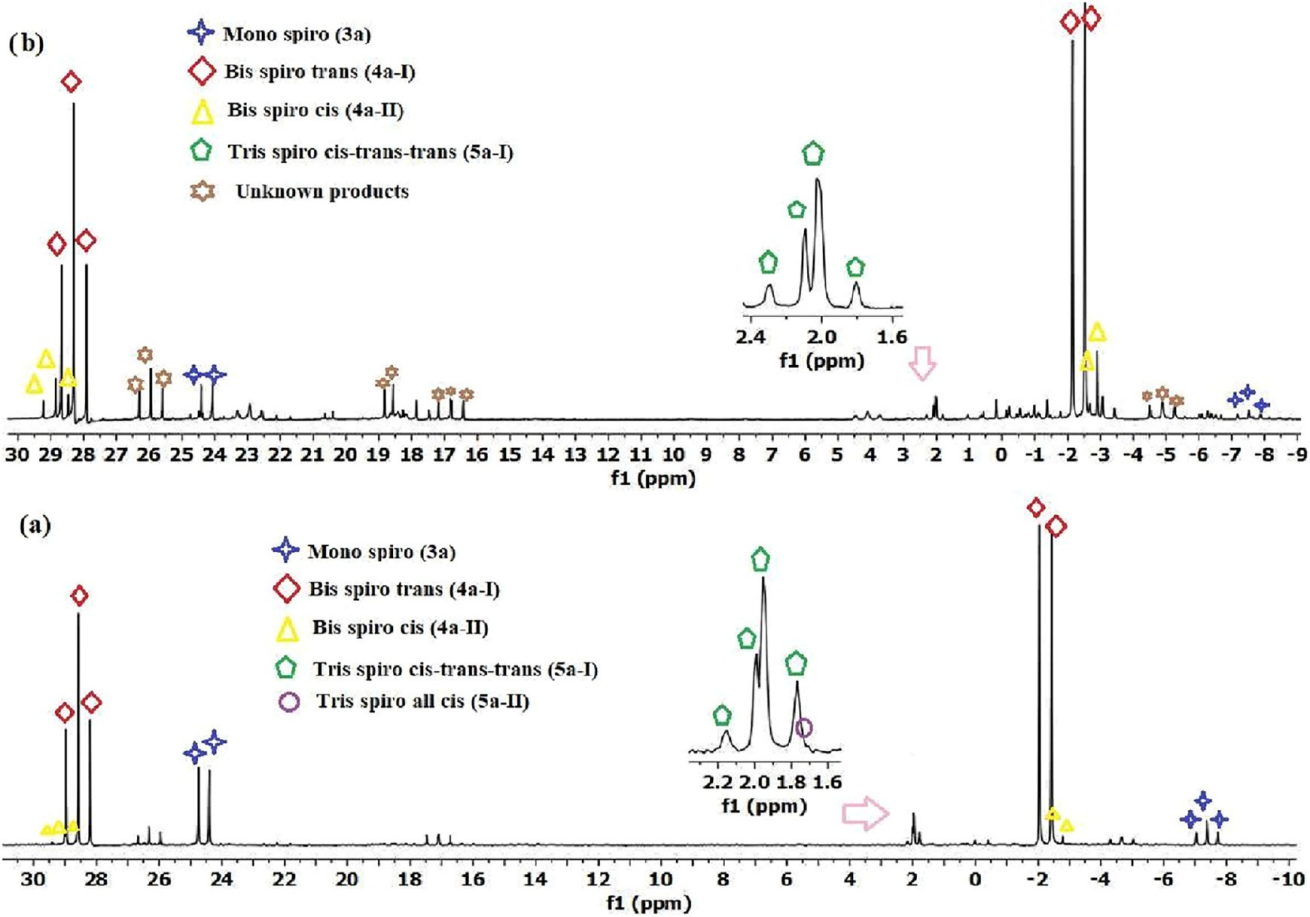
Proton-decoupled ^31^P­{^1^H} NMR spectrum of the reaction mixture of compound **1** with **2a** in a 1:2.2 mol ratio in THF solution with (a) NaH base, (b) Et_3_N base; the reaction mixture was filtered, and the solvent removed before dissolving in CDCl_3_ solution.

**Figure 3:**
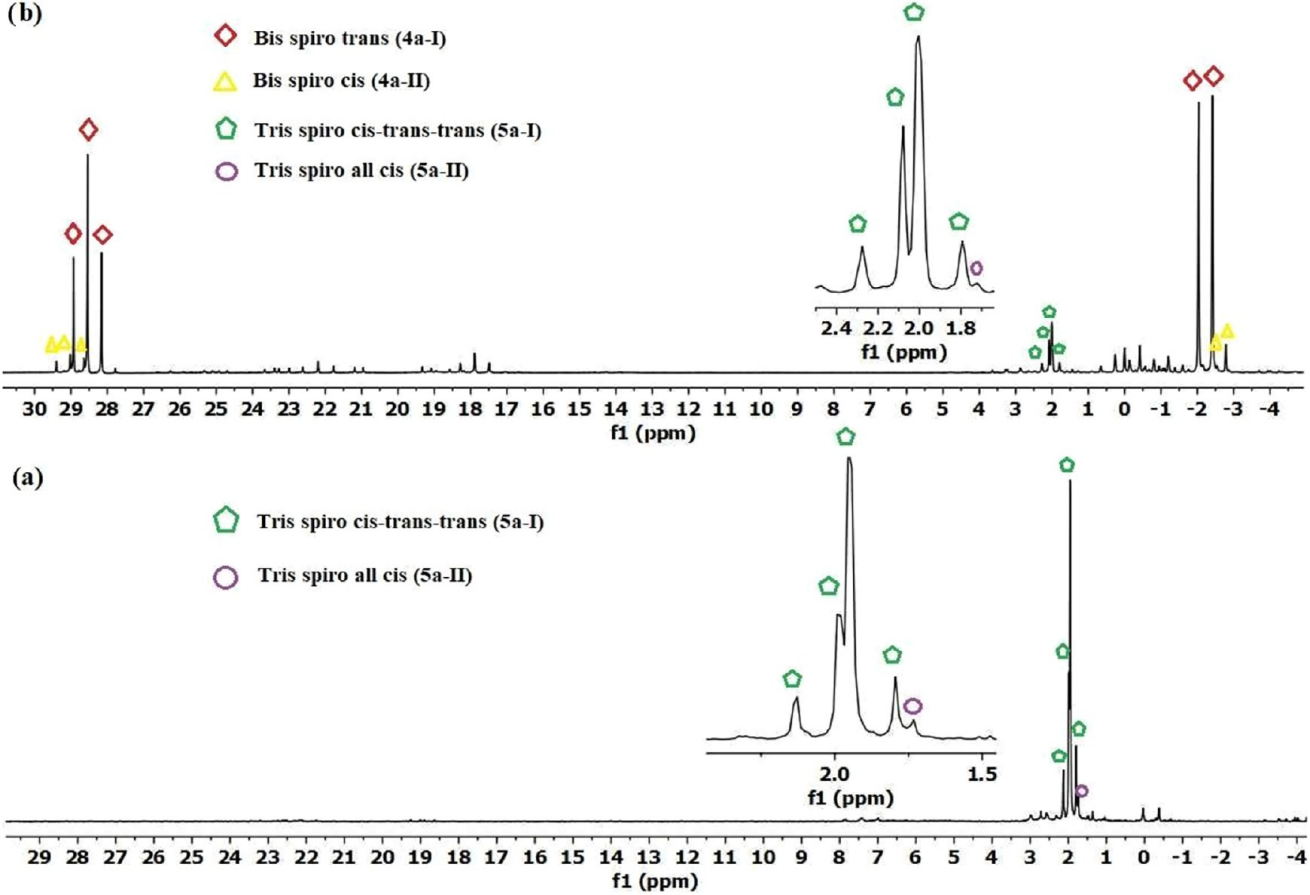
Proton-decoupled ^31^P­{^1^H} NMR spectrum of the reaction mixture of compound **1** with **2a** in a 1:3.2 mol ratio in THF solution with (a) NaH base (excess), (b) Et_3_N base; the reaction mixture was filtered, and the solvent removed before dissolving in CDCl_3_ solution.

**Figure 4:**
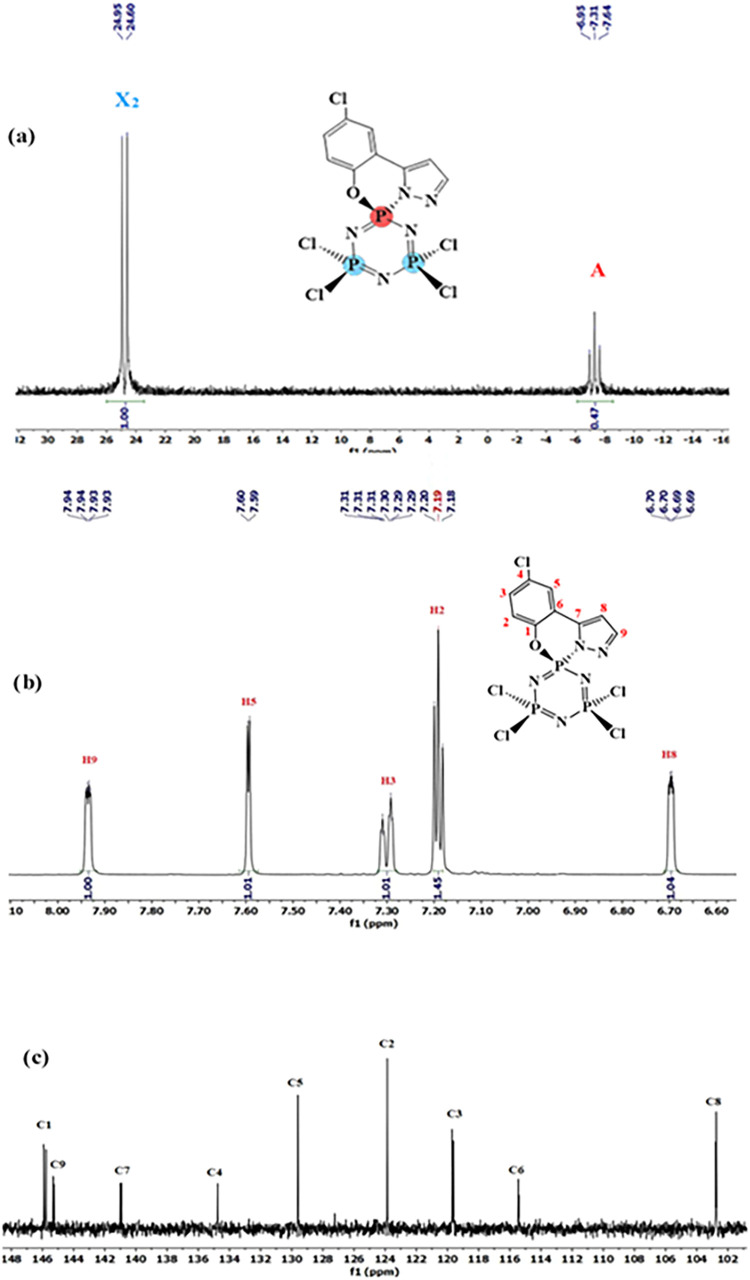
^31^P {^1^H} NMR spectrum (a) ^1^H NMR spectrum (b) ^13^ C NMR spectrum (c) of compound **3a**.

**Figure 5:**
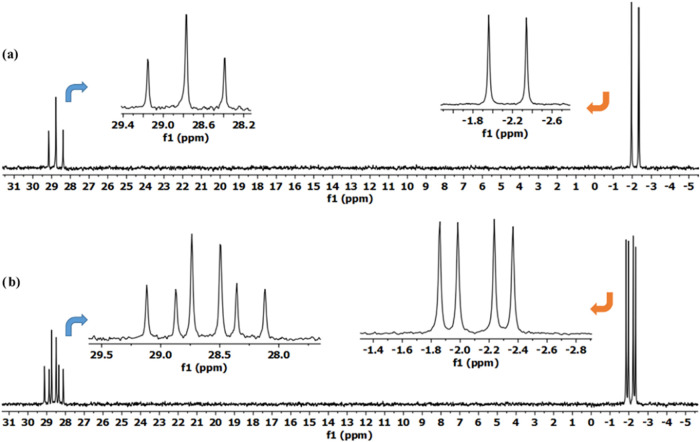
^31^P {^1^H} NMR spectrum of (**a**) compound **4a-I** (**b**) after the addition of CSA at ca. 10:1 mol ratio in CDCl_3_.

**Figure 6:**
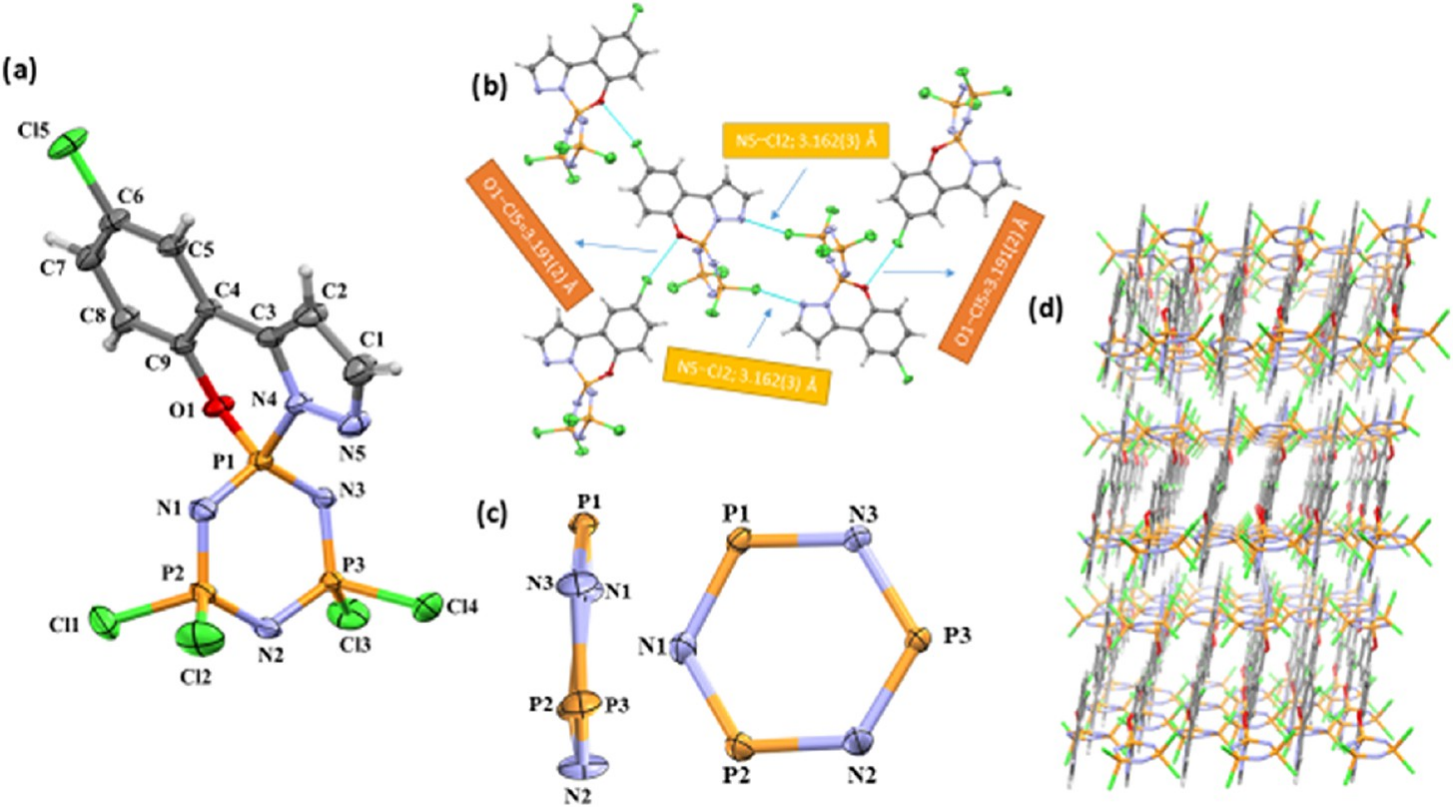
(a) The crystal structure of the compound **3a** with atom numbering scheme. Displacement ellipsoids drawn at the 30% probability level; (b) Some important intermolecular interactions in **3a**; (c) The conformations of the cyclotriphosphazene ring of **3a** (top and side view); (d) Perspective view of 3D supramolecular network viewed down the *b*-axis.

**Figure 7:**
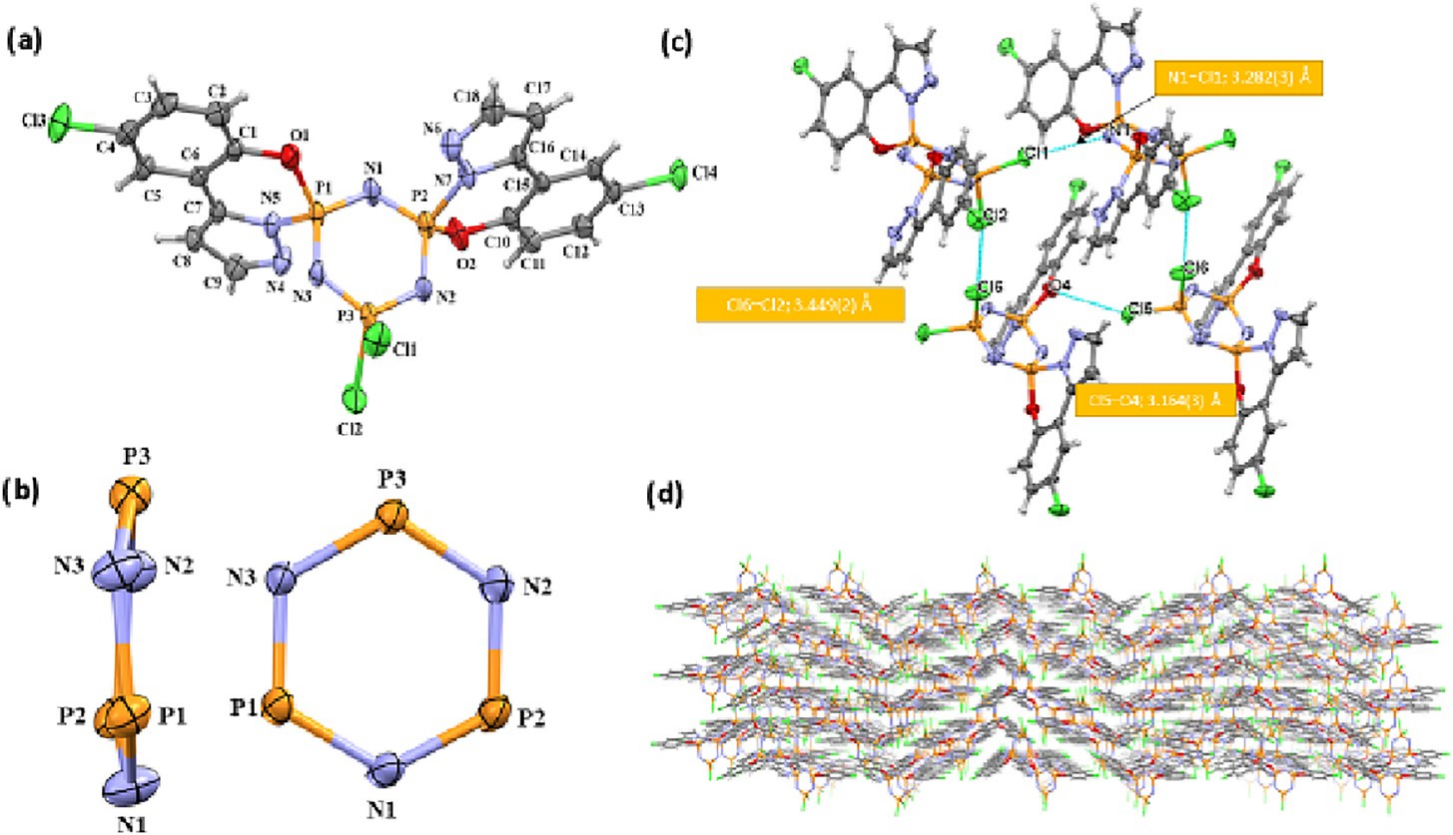
(a) The crystal structure of the compound **4a-I** with atom numbering scheme. Displacement ellipsoids drawn at the 30% probability level; (b) The conformations of the cyclotriphosphazene ring of **4a-I** (top and side view); (c) Some important intermolecular interactions in **4a-I**; (d) Perspective view of 3D supramolecular network viewed down the *b*-axis.

**Figure 8:**
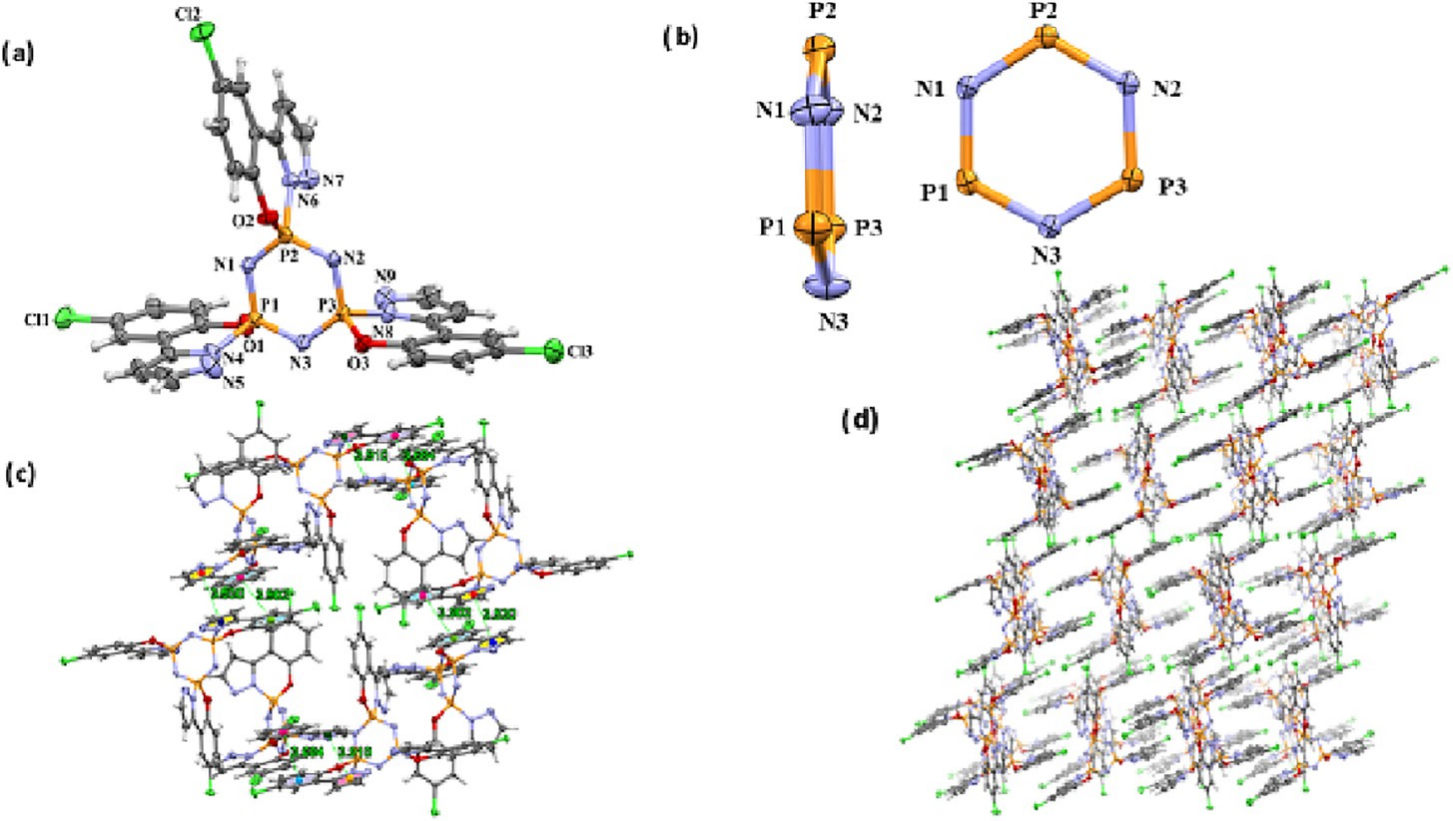
(a) The crystal structure of the compound **5a-I** with atom numbering scheme. Displacement ellipsoids drawn at the 30% probability level; (b) The conformations of the cyclotriphosphazene ring of **5a-I** (top and side view); (c) Some important intermolecular interactions in **5a-I**; (d) Perspective view of 3D supramolecular network viewed down the *b*-axis.

The *trans* dispiro compound **4a-I** consisting of two equal chiral centers were verified by ^31^P {^1^H} NMR spectroscopy using (*R*)-(−)-1-(9-anthryl)-2,2,2-trifluoroethanol as a chiral solvating agent (CSA),^23^ where splitting of the ^31^P {^1^H} NMR signals for **4a-I** was observed upon addition of the CSA in a 10:1 mol ratio (Figure 5). This splitting proved that **4a-I** is racemate (*RR*\*SS*), as expected.^23^

Examination of the ^31^P­{^1^H} NMR spectrum of the reaction between compounds **1** and **2a** in the presence of Et_3_N (1:1.2 mol ratio) revealed that 19% of the starting material (**1**) remained unreacted, while compound **3a** was obtained in 81% yield (Figure 1b). In the ^31^P­{^1^H} spectrum of the reaction mixture of HCCTP (**1**) and **2a** at a mole ratio of 1:2.2 in the presence of NaH base, compound **3a** (*ca*. 14%), compound **4a-I** (*ca*. 65%) and compound **4a-II** (*ca*. 4%.) were formed. Additionally, a different product (*ca*. 5%) with AB_2_ spin system was determined (Figure 2a), and mass analysis data after purification processes showed that three chloro phenol pyrazole groups are spiro-bound to HCCTP (**1**). Based on the *N* and *O* atoms in **2a**, it is likely that the structure is *cis*-*trans*-*trans* (**5a-I**) or *cis–cis–cis* (**5a-II**) isomers. In the case of a *cis–cis–cis* configuration, the ^31^P {^1^H} NMR spectrum would display an A_3_ spin system. However, the experimental data showed an AB_2_ pattern, which clearly indicates that the compound adopts a *cis*-*trans–trans* configuration (**5a-I**). This structural assignment was further confirmed by X-ray crystallographic analysis (Figure 8). The same products with different formation yield were observed in the ^31^P {^1^H} spectrum of the reaction mixture in the presence of the Et_3_N base at the same mole ratio (Figure 2b). The yields calculated based on the spectrum were **3a**; 6%, **4a-I**; 60%, **4a-II**; 11%, and **5a-I**; 3%, respectively. Finally, in the ^31^P {^1^H} NMR spectrum of the reaction of **1** and **2a** with NaH base at a 1:3.2 mol ratio, it was determined that compound **5a-I** was formed in 90% yield, and the all-*cis* isomer **5a-II** was formed in trace amount (2%) (Figure 3a). However, the reaction carried out in the presence of Et_3_N base showed greater product diversity (Figure 3b). The calculated values were **4a-I**; 56%, **4a-II**; 6%, **5a-I**; 19%, and **5a-II**; (trace amount), respectively. In our previous study, it was determined that the bonding patterns on the cyclotriphosphazene ring changed because of the tautomers formed by different *N* (*N* ^1^ and/or *N* ^2^) atoms.^16^ The chloro phenol pyrazole (**2a**) reactions proceeded through the *N* ^2^ atom, which is the only spiro binding type, although two different bases were used in the reaction procedures. Since NaH was a strong base, it appeared that the yield of the product formation is more proportional to the mole ratios, and that ligand **2a** increased the tendency to react.

The synthesis part of the experimental section contains the ^13^C and ^1^H NMR spectral data (chemical shifts, multiplicities, and coupling constants) of **3a**, **4a-I**, and **5a-I** (Figure 4, Figures S2–S3 and Figures S5 and S6), The H_8_ and H_9_ protons on the pyrazole ring are split multiple due to coupling with each other and with the phosphorus atom. While the H_9_ proton resonates in the range of 7.94–8.00 ppm, the H_8_ proton resonates in the range of 6.64–6.71 ppm. In compound **5a-I**, which has *cis–trans–trans* isomers, it can be seen that two of the three H_8_ protons are in the same position, while the other is different (Figure S5). While the H_5_ proton in the chloro phenol ring resonates at 7.59 and 7.58 ppm in compounds **3a** and **4a-I**, respectively, the peaks are split due to a long-range couplings of 2.4 and 2.5 Hz with the H_3_ proton, four bonds away. In compound **5a-I**, the H_5_ proton is split into multiple peaks at 7.52 ppm. In compounds **3a** and **4a-I**, the H_3_ and H_2_ protons resonated as doublets in the range of 7.18–7.31 ppm due to vicinal couplings (averaging 8.7 Hz). The integral values of the ^1^H NMR data for all compounds are consistent with the number of protons on the molecule. When examining the ^13^C NMR spectra, it is generally observed that the C_8_ and C_9_ carbon atoms on the pyrazole ring resonate at around 102.56 and 145.43 ppm, respectively, while the ipso C7 carbon on the pyrazole ring resonates at an average of 141.11 ppm (Figures S3 and S6). The other ipso carbons C_1_, C_4_, and C_6_ resonated at an average of 145.62, 134.92, and 115.67 ppm, respectively. The carbon atoms at positions C_2_, C_3_, and C_5_ in the chloro phenol ring are observed to resonate within the range of 119.84 ppm to 127.77 ppm (Figure 4, Figures S3 and S6).

### 3.2 Crystal Structure Analysis of the Compounds 3a, 4a-I and 5a-I by Single Crystal X-ray Diffraction

Single crystal X-ray diffraction technique is used to clarify the compounds’ crystal structures. Figures 6–8 shows the ORTEP diagrams and the atom-numbering systems. Table 3 additionally provides crystallographic data and refinement parameters details. Table 4 provides the crystals’ bond lengths and angles (**3a**, **4a-I** and **5a-I**).

**Table 3:** X-ray Crystallographic Data and Refinement Parameters for the Compounds **3a, 4a-I** and **5a-I**

	**3a**	**4a-I**	**5a-I**
empirical formula	C_9_H_5_Cl_5_N_5_OP_3_	C_18_H_10_Cl_4_N_7_O_2_P_3_	C_27_H_15_Cl_3_N_9_O_3_P_3_
formula weight	469.34	591.04	712.74
temperature/K	296.15	296.15	296.15
crystal system	monoclinic	monoclinic	monoclinic
space group	*P*2_1_/*c*	*P*2_1_/*n*	*C*2/*c*
*a*/Å	14.021(2)	7.8649(11)	24.787(8)
*b*/Å	15.609(3)	37.715(5)	13.745(4)
*c*/Å	7.9812(13)	15.924(2)	19.475(6)
α/deg	90	90	90
β/deg	95.963(3)	102.960(2)	102.502(4)
γ/deg	90	90	90
volume/Å^3^	1737.2(5)	4603.3(11)	6478(3)
*Z*	4	8	8
ρ_calc_ g/cm^3^	1.795	1.706	1.462
μ/mm^–1^	1.118	0.757	0.476
*F*(000)	928.0	2368.0	2880.0
crystal size/mm^3^	0.231 × 0.136 × 0.116	0.196 × 0.145 × 0.092	0.183 × 0.091 × 0.064
Radiation	Mo Kα (λ = 0.71073)	Mo Kα (λ = 0.71073)	Mo Kα (λ = 0.71073)
2Θ range for data collection/deg	2.92 to 54.958	2.16 to 54.97	3.366 to 50
Reflections collected	25875	52622	26610
Independent reflections	3974 [*R*_int_ = 0.0433, *R*_sigma_ = 0.0263]	10535 [*R*_int_ = 0.0532, *R*_sigma_ = 0.0581]	5702 [*R*_int_ = 0.0981, *R*_sigma_ = 0.0976]
data/restraints/parameters	3974/0/208	10535/0/613	5702/0/406
goodness-of-fit on *F* ^2^	1.044	1.059	0.994
final *R* indexes [*I* ≥ 2σ (*I*)]	R1 = 0.0413, wR2 = 0.1031	R1 = 0.0601, wR2 = 0.1128	R1 = 0.0592, wR2 = 0.1276
final *R* indexes [all data]	R1 = 0.0528, wR2 = 0.1111	R1 = 0.1076, wR2 = 0.1265	R1 = 0.1323, wR2 = 0.1440
largest diff. peak/hole/e Å^–3^	0.54/–0.56	0.42/–0.42	0.22/–0.38

**Table 4:** Some Bond and Conformational Parameters of the Compounds **3a, 4a-I** and **5a-I**

	**3a**	**4a-I**	**5a-I**
P1–N1	1.572(2)	1.564(3)	1.568(3)
P1–N3	1.577(2)	1.577(3)	1.571(3)
P2–N1	1.569(2)	1.562(3)	1.571(3)
P2–N2	1.572(2)	1.570(3)	1.576(3)
P3–N2	1.571(2)	1.575(3)	1.581(3)
P3–N3	1.569(2)	1.571(3)	1.572(3)
N1–P1–N3	117.59(11)	117.73(17)	117.80(17)
N1–P2–N2	119.22(12)	117.51(17)	116.69(17)
N2–P3–N3	118.59(12)	118.18(18)	118.04(17)
P1–N1–P2	121.00(13)	122.6(2)	123.2(2)
P2–N2–P3	120.10(15)	121.5(2)	122.29(19)
P1–N3–P3	122.03(14)	120.87(19)	121.5(2)
P1–O1	1.5708(19)	1.572(3)	1.581(3)
P1–N4	1.675(2)		1.683(4)
P1–N5		1.683(3)	
P2–O2		1.568(3)	1.584(3)
P2–N7		1.674(3)	
P2–N6			1.692(3)
P3–O3			1.581(3)
P3–N8			1.671(4)
O1–P1–N4	98.82(10)		98.28(17)
O1–P1–N5		99.12(15)	
O2–P2–N6		99.84(15)	98.96(16)
O3–P3–N8			99.15(17)
Puckering amplitude, Q for P_3_N_3_ ring	0.1285(19)	0.142(3)	Planar
Max. Deviation for P_3_N_3_ ring	–0.0863(10) (P2)	0.085(3) (N3)	0.047(4) (N3)

Compound **3a** crystallizes in the monoclinic space group *P*2_1_/*c* with one molecule in the asymmetric unit (*Z*′ = 1), corresponding to four formula units per unit cell (*Z* = 4). In a crystal structure, the Z value corresponds to the general position multiplicity multiplied by the number of independent molecules in the asymmetric unit (Z′).^32^ In compound **4a-I**, two crystallographically independent molecules in the asymmetric unit (*Z*′ = 2). Each independent molecule is generated by symmetry to give 4 copies (2 × 4 = 8 molecules per unit cell). Centering and symmetry operations generate 8 molecules per unit cell (*Z* = 8). In compound **5a-I** has C-centered monoclinic space group (*C*2/*c*). The general position multiplicity is 8. One independent molecule is in the asymmetric unit (*Z*′ = 1). Centering and symmetry operations generate 8 molecules per unit cell (Table 3). All crystal structures contain a 6-membered cyclotriphosphazene skeleton and the chlorophenol pyrazol group is spiro-fused to the P_3_N_3_ ring. Figure 6a shows that in the crystal structure of compound **3a**, a chloro phenol pyrazole group is spiro-bonded to the P1 phosphorus atom. The puckering parameter^33^ of [P1/N1/P2/N2/P3/N3] are in slightly twisted configuration with the phosphazene ring, N_3_P_3_ (Figure 6c); *Q*_T_ = 0.1387(12) Å, φ = 299.2(9), θ = 107.1(8) (for **3a**) The distances of the N1, N2, and N3 atoms in the N_3_P_3_ rings from the best planes of the phosphorus (P1, P2, and P3) atoms are 0.052(2) Å, 0.050(3) Å, and −0.056(3) Å (for **3a**), respectively. For single-crystal X-ray analysis of **4a-I**, the chloro phenol pyrazole groups are spiro-bonded to the P1 and P2 phosphorus atoms, and *N* and *O* atoms in the pyrazole derivative are in a *trans* position relative to each other (Figure 7a). The *trans* **4a-I** molecule, which has two equivalent chiral centers on the P1 and P2 phosphorus atoms, could potentially produce two racemates (*RR*/*SS*) of optically active isomers.^16^ According to the results of the ^31^P­{^1^H} NMR spectroscopy on addition of the chiral solvating agent (CSA) analysis, the **4a-I** molecule is racemic (*RR*/*SS*) (Figure 5). Despite having two stereogenic centers, the molecule crystallizes as a racemic mixture and its crystal structure was identified in the centrosymmetric space group *P*2_1_/*n*. Because of this, the crystal is achiral and the X-ray diffraction data cannot reveal the exact arrangement of the chiral centers.^34^ Therefore, there is no reported absolute stereochemical assignment (P1 and P2 phosphorus atoms; *RR* or *SS*) in checkcif file.

In **5a-I** crystal structure, the chloro phenol pyrazole groups spiro-bonded to all three phosphorus atoms are in the *cis–trans*-*trans* position relative to each other (at the *N* and *O* positions) (Figure 8a). The cyclotriphosphazene rings in compounds **4a-I** (Figure 7b) and 5**a-I** (Figure 8b) are planar and slightly twisted, respectively. Table 4 shows that the puckering amplitude value of *Q*_T_ is 0.142(3), with the highest deviation from the main plane is 0.085(3) (N3) for **4a-I** and 0.047(4) (N3) for **5a-I**. The endocyclic P–N bond lengths for compound **3a** range from 1.569(2) Å to 1.577(2) Å [P1–N1; 1.572(2) Å, P1–N3; 1.577(2) Å, P2–N1; 1.569(2) Å, P2–N2; 1.572(2) Å, P3–N2; 1.571(2) Å, P3–N3; 1.569(2) Å], for **4a-I**, they range from 1.562(3) Å to 1.577(3) Å [P1–N1; 1.564(3) Å, P1–N3; 1.577(3) Å, P2–N1; 1.562(3) Å, P2–N2; 1.570(3) Å, P3–N2; 1.575(3) Å, P3–N3; 1.571(3) Å], and for **5a-I**, they range from 1.568(3) Å to 1.581(3) Å [P1–N1; 1.568(3) Å, P1–N3; 1.571(3) Å, P2–N1; 1.571(3) Å, P2–N2; 1.576(3) Å, P3–N2; 1.581(3) Å, P3–N3; 1.572(3) Å] (Table 4). It is observed that the exocyclic P–N bond lengths are longer (P1–N4; 1.675(2) Å for **3a**, P1–N5; 1.683(3) Å and P2–N7; 1.674(3) Å for **4a-I**, P1–N4; 1.683(4) Å, P2–N6; 1.692(3) Å and P3–N8; 1.671(4) Å for **5a-I**) than the endocyclic P–N bond angles. This may be due to the single and double bond arrangement between the P–N bonds in the HCCTP skeleton.^35^ The average P–N–P bond angles are 121.04° for **3a**, 121.66° for **4a-I**, and 122.63° for **5a-I**, respectively. The N–P–N bond angles are smaller than the P–N–P angles, being 118.47°, 117.81°, and 117.51° for **3a**, **4a-I**, and **5a-I**, respectively (Table 2). HCCTP (**1**) has equivalent P–N bond lengths of 1.581 Å (σ 0.004–0.005 Å) in its crystal structure. The P–Cl bond lengths range from 1.991 Å to 1.995 Å (σ 0.004 to 0.005 Å).The mean P–N–P angle (121.4°) is substantially larger than the mean N–P–N angle (118.4°) in the ring.^36^ The endocyclic and exocyclic bond lengths and angles of the compounds are influenced by the ring conformations, the steric hindrances of the substituents, the negative hyper-conjugation, and the kind of groups on the phosphorus atoms.^37,38^ The bond lengths and angles are comparable to those of other cyclotriphosphazene derivatives with a phenol pyrazole group, except for minor differences that may arise based on the kind of group and/or bonding arrangement.^16,23^

The primary interactions in crystal packing are hydrogen bonds and van der Waals contacts, and intermolecular interactions less than 3.5 Å are essential for preserving the crystal structure. There are no classical hydrogen bonds in any of the three crystal structures. In compound **3a**, two significant intermolecular interactions are observed between Cl–N and Cl–O atoms [Cl2···N5; 3.162(3) Å and Cl5···O1; 3.191(2) Å] (Figure 6b). The crystal structure of **4a-I** includes intermolecular interactions such as Cl–N, Cl–Cl, Cl–H, Cl–C, and N–H. Cl1···N1; 3.282(3) Å, Cl2···C17; 3.336(5) Å and Cl2···Cl6; 3.449(2) Å intermolecular interactions (Figure 7c).

Again, Y-X···Cg (C13–Cl4···Cg15; 3.571 (2) Å and C31–C18···Cg2; 3.438 (2) Å) and π-π interactions play role in the packing of crystals. The Cg2-Cg20; 3.889(2) Å, Cg6-Cg19; 3.767(3) Å, and Cg7-Cg15; 3.874(2) Å interactions are π-π interactions between the pyrazole and phenyl rings or between the phenyl rings. (Cg2 and Cg20 are the centroids of the N6–N7–C16–C17-C18 and C28–C33 rings, Cg6 and Cg19 are the centroids of the C1–C6 and C19–C24 rings, Cg7 and Cg15 are the centroids of the N13–N14–C34–C35-C36 and C10–C15 rings, respectively). In **5a-I**, although many intermolecular interactions in the crystal structure are formed between H and N and between H- Cl atoms, Cl1···C25; 3.433(5) Å and Cl1^**...**^N1; 3.162(4) Å interactions are also included. π-π interactions and X-H···Cg in crystal packing (C2–H2···Cg9; X···Cg; 3.639 (5) Å) interactions are seen to make a significant contribution. Cg3···Cg3; 3.531(3) Å, Cg1···Cg3; 3.910(3) Å, Cg8···Cg10; 3.684(3) Å and Cg10···Cg10; 3.902(3) Å are important π-π interactions (Cg3 is the centroid of the N8–N9–C19–C20-C21, Cg1 and Cg3 are the centroids of the N4–N5–C9–C8-C7 and N8–N9–C19–C20-C21 rings, Cg8 and Cg10 are the centroids of the C1–C6 and C22–C27 rings, respectively) (Figure 8c). Intermolecular interactions and π-π stacking may contribute to the packing of molecules and stabilize the crystal structure in the crystalline structures (**3a**, **4a-I** and **5a-I**). Figures 6–8d display the perspective views of each crystal’s 3D supramolecular network.

### 3.3 Thermal Properties of Phenol Pyrazole Cyclotriphosphazene Derivatives

Cyclotriphosphazene-based compounds are of the utmost importance due to the presence of both nitrogen and phosphorus atoms in their structure, as well as their ability to undergo nucleophilic substitution reactions, allowing for tunable functionalities. Cyclotriphosphazenes are encountered in several fields of application, such as flame retardants and lubricants, owing to their notable thermal stability and fire-resistance properties.^39,40^ Due to their inherently rigid and thermally stable cyclic phosphorus–nitrogen backbones, combined with the electron-withdrawing chlorine substituents, novel cyclotriphosphazene derivatives are anticipated to demonstrate enhanced thermal performance. For this reason, the thermal properties of the cyclotriphosphazene-based compounds were examined via thermogravimetric analysis (TGA). TGA thermograms of all phenol pyrazole cyclotriphosphazene derivatives (**3a**, **3b**, **4a-I**, **4b-I**, **5a-I**, and **5b-I**) were obtained between room temperature and 700 °C at a heating rate of 10 °C/min under 50 mL/min N_2_ flow (Figure 9). Weight losses as a function of temperature were recorded, and parameters such as the residual mass at 700 °C and the number of thermal decomposition steps were evaluated for each compound. The data clearly showed that the introduction of a chlorine substituent on the phenol-pyrazole ring made cyclotriphosphazene compounds more resistant to thermal decomposition, enhancing the char residues of **3a** (24.8%), **4a-I** (68.6%), and **5a-I** (60.3%), compared to chlorine-free cyclotriphosphazene derivatives (**3b**, **4b-I**, and **5b-I**). Furthermore, the increase in chlorine substituents influenced the decomposition pathways of cyclotriphosphazene compounds; both **4a-I** and **5a-I** decomposed in two steps, while **3a** underwent a single-step decomposition. Chlorine-free cyclotriphosphazene derivatives (**3b**, **4b-I** and **5b-I**) decomposed in a single step, with corresponding char residues of 19.4%, 42.4% and 21.3%, respectively.

**Figure 9:**
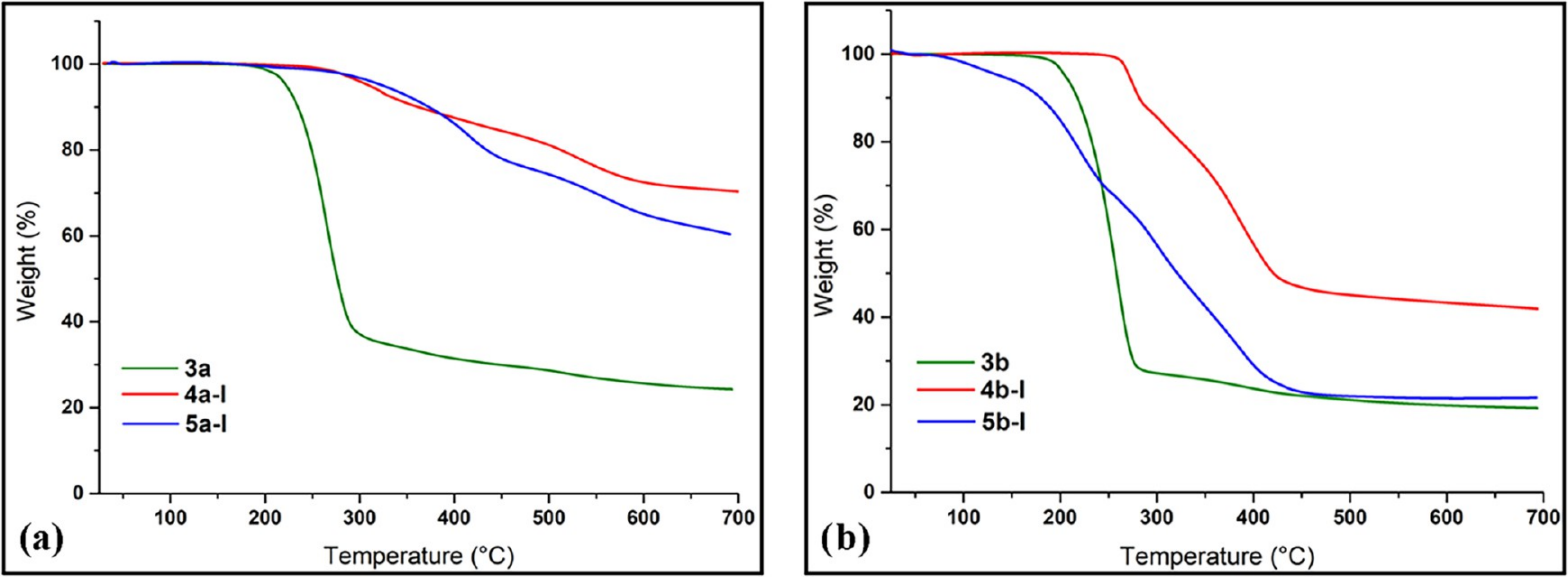
TGA curves of compounds (a) **3a**, **4a-I** and **5a-I**; (b) **3b**, **4b-I** and **5b-I**.

### 3.4 Biologic Activity

In both PANC-1 and MIA-PaCa-2 cell lines, 24-h treatment with compound **3a** significantly reduced cell viability in a dose-dependent manner, as demonstrated by MTT and colony formation assays (Figure 10a,c). Statistical analysis revealed IC_50_ values of 24.57 μg/mL and 23.30 μg/mL for PANC-1 and MIA-PaCa-2 cells, respectively (Figure 10b). Based on the molecular weight of **3a** (469.34 g/mol), these correspond to approximately 52.35 μM (24.57 μg/mL) in PANC-1 cells and 49.64 μM (23.30 μg/mL) in MIA PaCa-2 cells.

**Figure 10:**
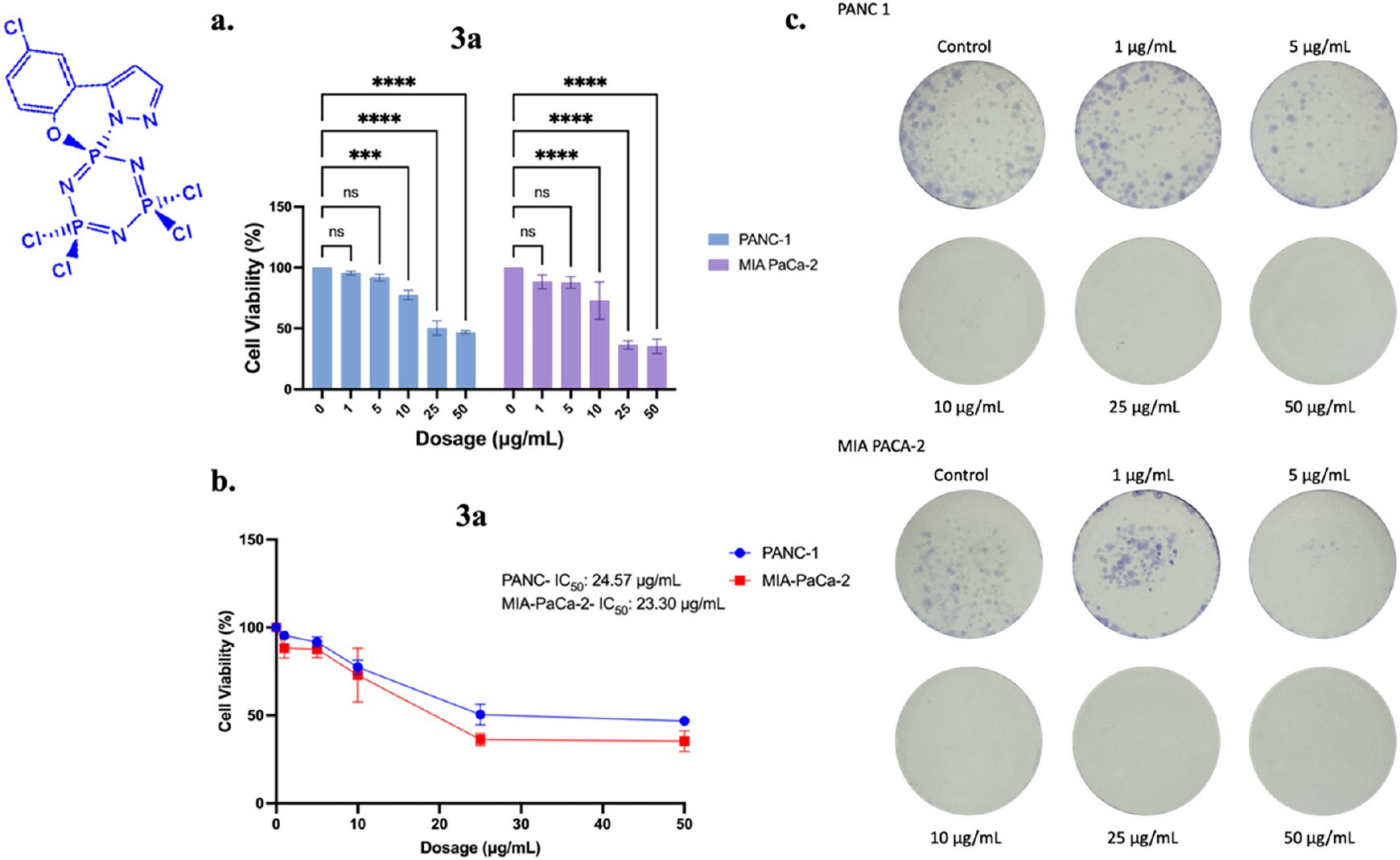
(a) MTT assay (Data represent mean values of four technical and three biological replicates, with error bars indicating standard deviation. Statistical analysis was performed using two-way ANOVA with Dunnett’s post-test (ns *p* > 0.5, ***p < 0.001, ****p < 0.0001). (b) IC_50_ values calculated from MTT assay, and (**c)** colony formation assay in PANC-1 and MIA-PaCa-2 cells following 24-h treatment with compound **3a**.

Gemcitabine, a standard chemotherapeutic used in pancreatic cancer, has been reported to inhibit PANC-1 cell viability at 24 h with an IC_50_ of 4.12 μM (1.08 μg/mL),^41^ and submicromolar activity has also been reported for MIA PaCa-2 at 24 h (e.g., ∼0.76 μM (0.20 μg/mL)).^42^ However, compound screening assays are commonly performed at fixed concentrations in the low-micromolar range (typically 1–10 μM), while compounds exhibiting IC_50_ values below ∼100 μM are often considered to show potentially relevant in vitro activity and may be prioritized for further optimization in early stage screening campaigns.^43^

Notably, **3a** represents a hybrid inorganic–organic scaffold in which a pyrazole pharmacophore is integrated onto a spiro-cyclotriphosphazene core, providing a modular platform for structure diversification while already achieving clear growth inhibition in both pancreatic cancer models within 24 h.

Fluorescence microscopy analysis of compound **3a** revealed that treatment of cells with increasing concentrations of the compound (1, 5, 10, 25, and 50 μg/mL) resulted in a dose-dependent decrease in cell viability and mitochondrial function (Figure 11). In the control group, strong green fluorescence from DCFDA indicated higher ROS levels, while robust red (MitoTracker) and blue (DAPI) staining indicated healthy mitochondria and numerous intact nuclei. As the concentration of the compound increased, green fluorescence decreased progressively, suggesting either diminished ROS production or fewer viable cells. Similarly, MitoTracker and DAPI signals declined, indicating compromised mitochondrial integrity and reduced cell numbers at higher doses. The merged images further highlighted these effects, showing a marked reduction in overall fluorescence intensity and overlap, particularly at 25 and 50 μg/mL, consistent with significant cytotoxicity and cellular health loss.

**Figure 11:**
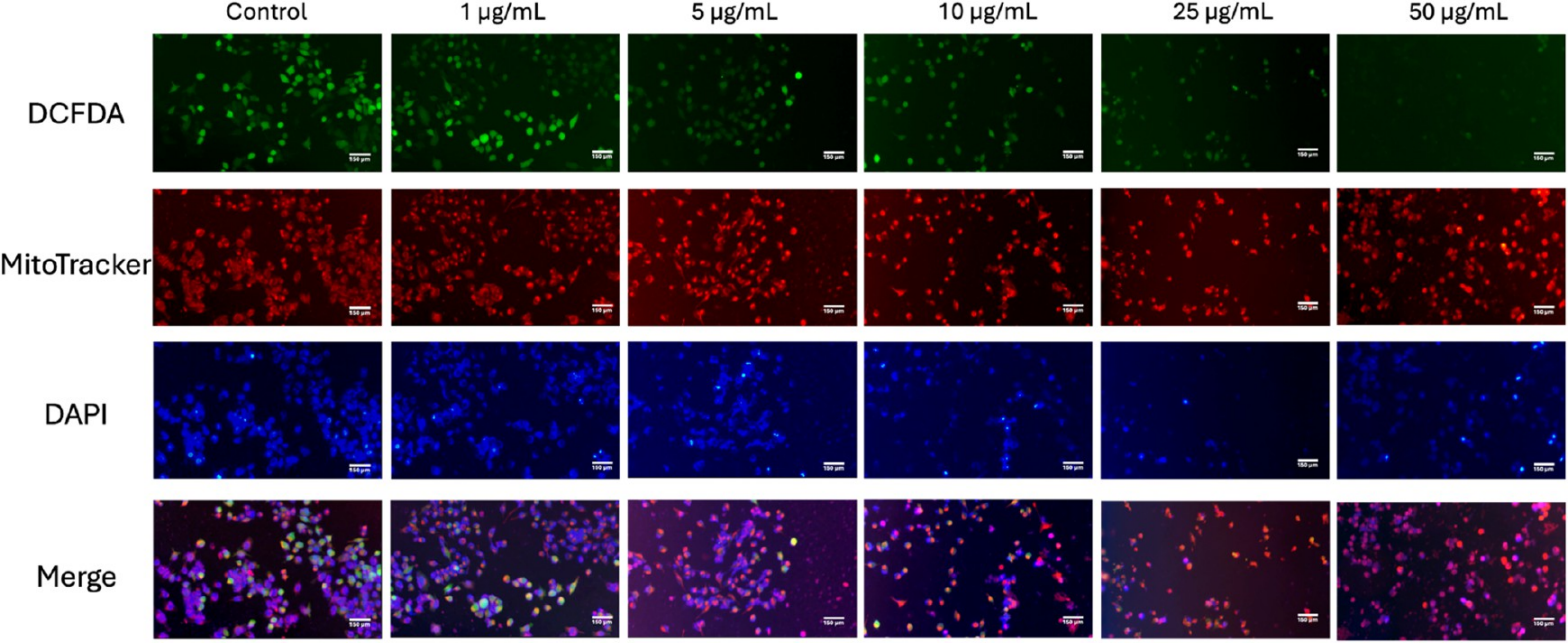
Fluorescence microscopy images of PANC-1 cells after 24-h treatment with **3a**, staining of H_2_-DCFDA (green, ROS), MitoTracker (red, mitochondria), and DAPI (blue, nuclei), along with their merged image. Scale bar is 150 μm.

Treatment of MIA-PaCa-2 with increasing concentrations of the compound **3a** (1, 5, 10, 25, and 50 μg/mL) for 24 h resulted in a concentration-dependent reduction in cellular health and viability, as observed by fluorescence microscopy (Figure 12). In control, strong green fluorescence from DCFDA indicated elevated ROS levels, while intense red (MitoTracker) and blue (DAPI) signals reflected healthy mitochondria and abundant intact nuclei. As the concentration of the compound increased, there was a progressive decrease in green fluorescence, suggesting a reduction in ROS production or a decline in the number of metabolically active cells. Similarly, both MitoTracker and DAPI staining intensities diminished, indicating mitochondrial dysfunction and reduced cell numbers at higher doses. The merged images further confirmed these findings, with a marked reduction in overall fluorescence and signal overlap at 25 and 50 μg/mL, consistent with significant cytotoxic effects and loss of cellular integrity at elevated concentrations.

**Figure 12:**
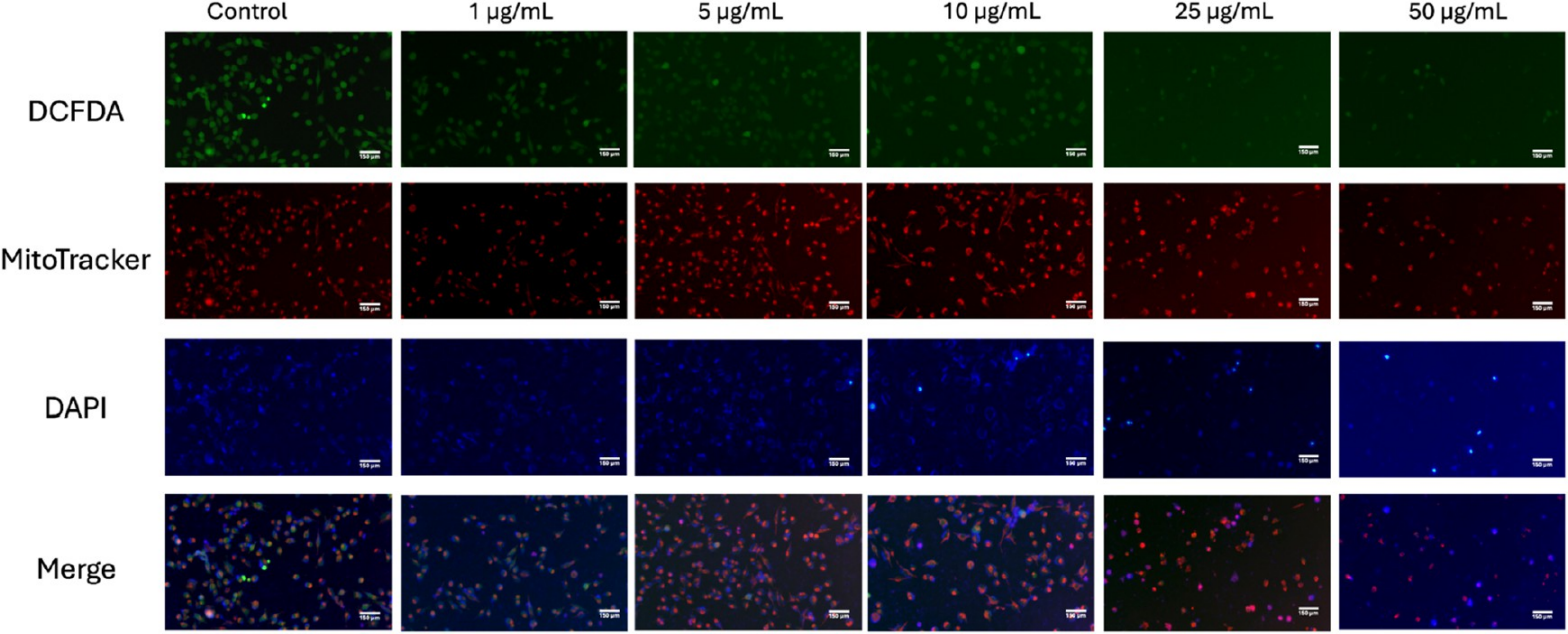
Fluorescence microscopy images of MIA-PaCa-2 cells after 24-h treatment with **3a**, staining of H_2_-DCFDA (green, ROS), MitoTracker (red, mitochondria), and DAPI (blue, nuclei), along with their merged image. Scale bar is 150 μm.

The effect of compound **4a-I** on cell viability was assessed in PANC-1 and MIA-PaCa-2 cells using MTT assays following 24-h treatment, with no statistically significant changes observed (Figure 13a). However, colony formation assays revealed dose-dependent inhibition of colony formation in PANC-1 cells at 5, 10, and 50 μg/mL compared with controls. In contrast, MIA-PaCa-2 cells showed reduced colony formation only at the highest tested dose (50 μg/mL) (Figure 13b).

**Figure 13:**
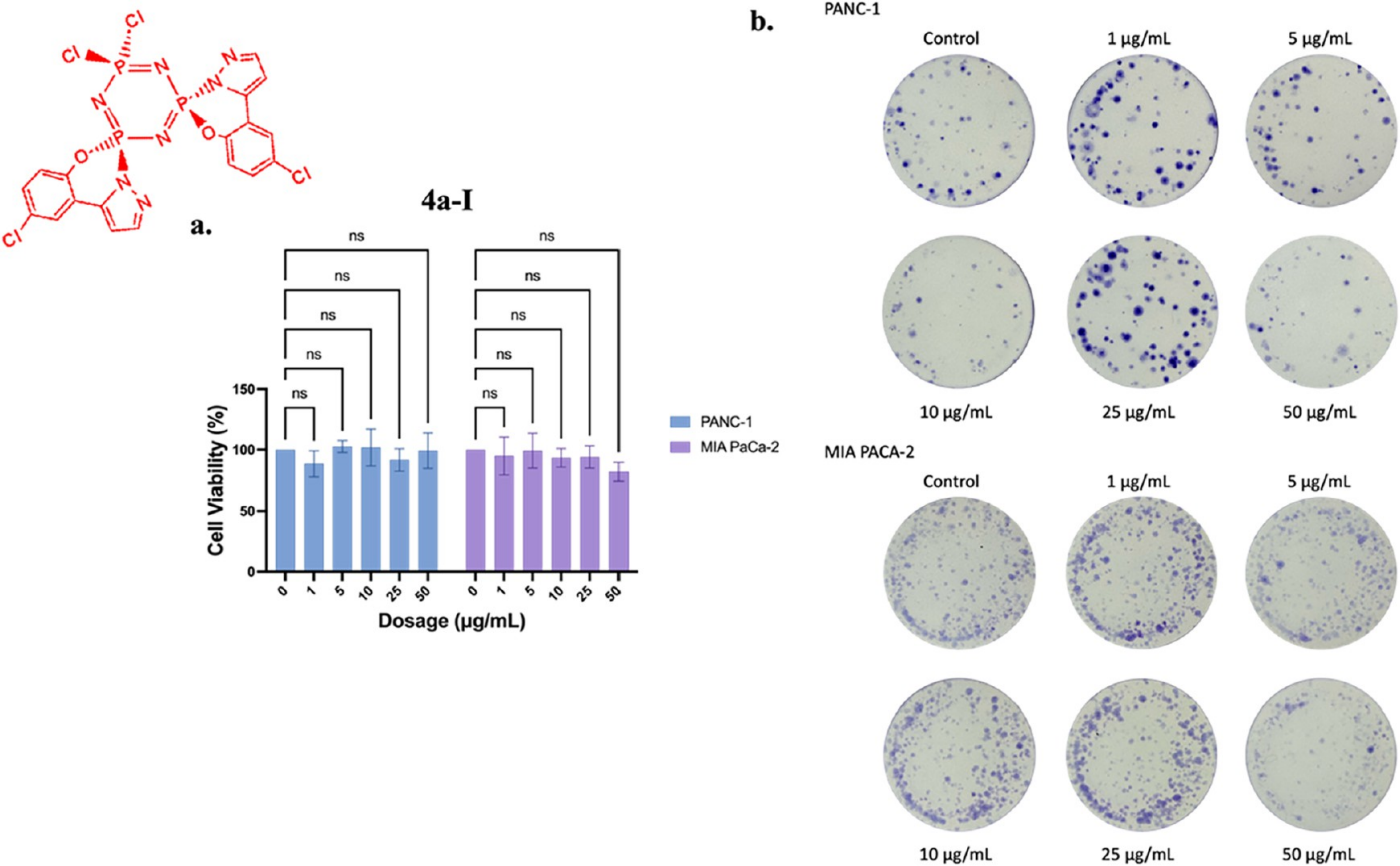
Effects of **4a-I** on (a) cell viability and (b) colony formation in PANC-1 and MIA-PaCa-2 cells following 24-h treatment. Data represents the MTT assay mean values ± standard deviation from four technical and three biological replicates. Two-way ANOVA determined statistical significance with Dunnett’s posthoc test (ns: *p* > 0.05).

Treatment of both PANC-1 (Figure S8) and MIA PaCa-2 (Figure S9) cells with increasing concentrations of **4a-I** (1, 5, 10, 25, and 50 μg/mL) for 24 h did not cause significant changes in oxidative stress, mitochondrial integrity, or nuclear morphology compared to untreated controls. Green fluorescence from DCFDA staining, which indicates intracellular reactive oxygen species, remained consistently low across all concentrations in both cell lines, suggesting no induction of oxidative stress. Similarly, red fluorescence from MitoTracker staining showed stable mitochondrial labeling, and blue fluorescence from DAPI indicated a comparable number and distribution of nuclei in both treated and control groups. The merged images further confirmed these observations, displaying persistent colocalization of mitochondrial and nuclear signals without evidence of cytotoxicity or significant alterations in cell health at any tested concentration. Overall, these results demonstrate that **4a-I** does not induce oxidative stress, mitochondrial dysfunction, or significant cell death in PANC-1 or MIA PaCa-2 cells under the experimental conditions used.

Exposure of PANC-1 and MIA PaCa-2 pancreatic cancer cells to increasing concentrations of **5a-I** (1, 5, 10, 25, and 50 μg/mL) did not cause a notable reduction in cell viability, as determined by the MTT assay (Figure 14). Across all concentrations tested, cell survival in both cell lines remained nearly 100%, with no statistically significant differences compared to the untreated controls. These findings indicate that **5a-I** does not exhibit measurable cytotoxic or growth-inhibitory activity against these pancreatic cancer cells at doses up to 50 μg/mL.

**Figure 14:**
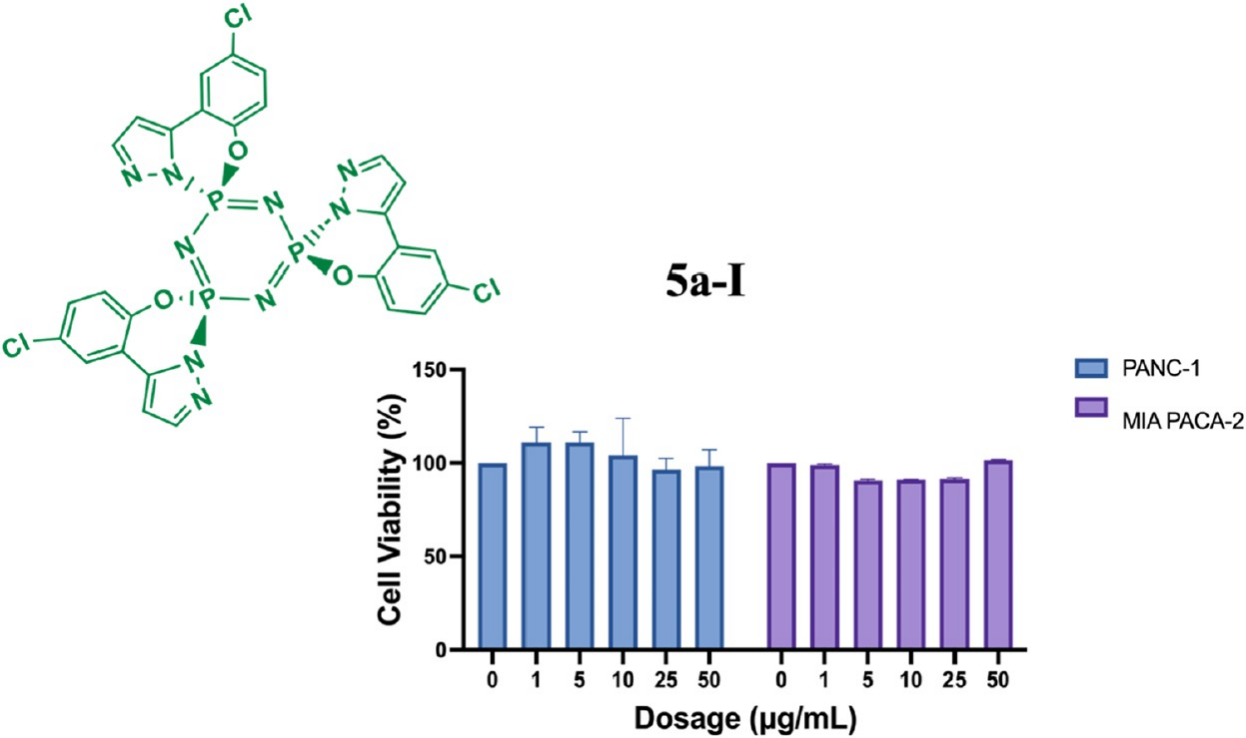
Effects of **5a-I** on cell viability in PANC-1 and MIA-PaCa-2 cells following 24-h treatment. Data represents the MTT assay mean values ± standard deviation from four technical and three biological replicates. Two-way ANOVA determined statistical significance with Dunnett’s posthoc test (ns: *p* > 0.05).

To evaluate the therapeutic properties of synthesized compounds, MTT cell viability and colony-forming assays were performed for each compound in a dose-dependent manner. Compound **3b** did not exhibit a significant cytotoxic effect on either cell line in a dose-dependent manner, as determined by the MTT assay (Figure 15a). The determination of mitochondrial activity by MTT assay was supported by the colonization capacity assay (Figure 15b). After the colony formation assay, the colonization capacity was inhibited in PANC-1 and MIA-PaCa-2 cells at a 5 μg/mL application dose. Still, an increase in the number of colonies was observed at a 25 μg/mL application dose. This compound, which was not observed to reduce cell viability in the MTT assay, reduced the colonization ability in both cell lines at application doses of 5, 10, and 50 μg/mL.

**Figure 15:**
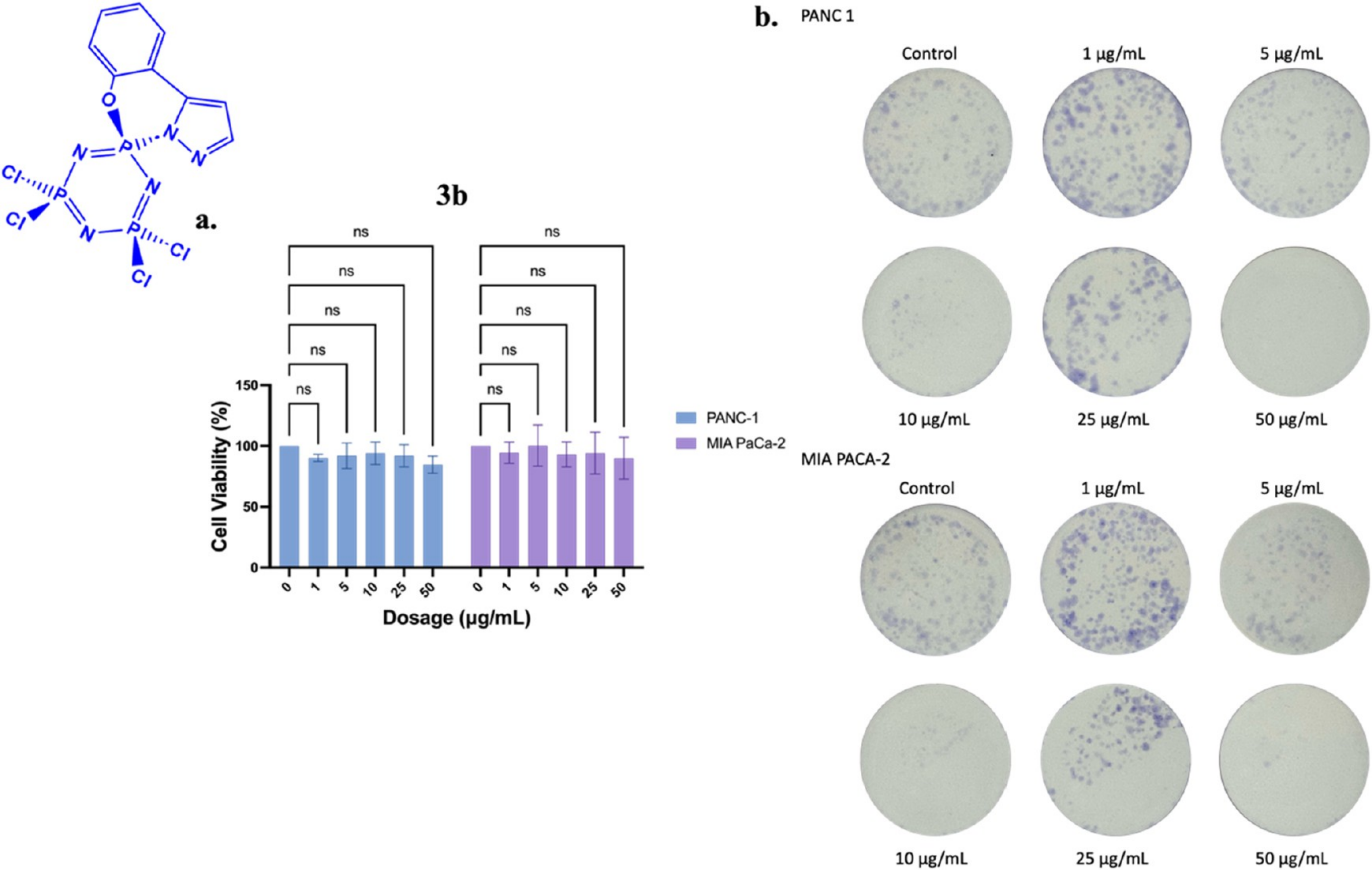
Effects of **3b** on (a) cell viability and (b) colony formation in PANC-1 and MIA-PaCa-2 cells following 24-h treatment. Data represents the MTT assay mean values ± standard deviation from four technical and three biological replicates. Two-way ANOVA determined statistical significance with Dunnett’s posthoc test (ns: *p* > 0.05).

Treatment of both PANC-1 (Figure S10) and MIA-PaCa-2 (Figure S11) cells with increasing concentrations of **3b** (1, 5, 10, 25, and 50 μg/mL) for 24 h did not result in significant alterations in oxidative stress, mitochondrial integrity, or nuclear morphology compared to untreated controls. Across all tested concentrations, the ROS level, as determined by DCFDA staining, remained consistently low, suggesting that **3b** did not induce oxidative stress in either cell line. Similarly, MitoTracker staining showed similar mitochondrial activity in the treated and control groups, and DAPI revealed comparable numbers and distributions of nuclei in both groups. The merged images further confirmed these findings, demonstrating persistent colocalization of mitochondrial and nuclear signals without evidence of cytotoxicity or significant changes in cell health at any dose. Overall, these results indicate that **3b** does not provoke oxidative stress, mitochondrial dysfunction, or significant cell death in PANC-1 or MIA PaCa-2 cells under the experimental conditions used. While compound **4b-I** exhibited no cytotoxic effects in either cell line, the clonogenic assays revealed no effects on colony formation capacity (Figure 16).

**Figure 16:**
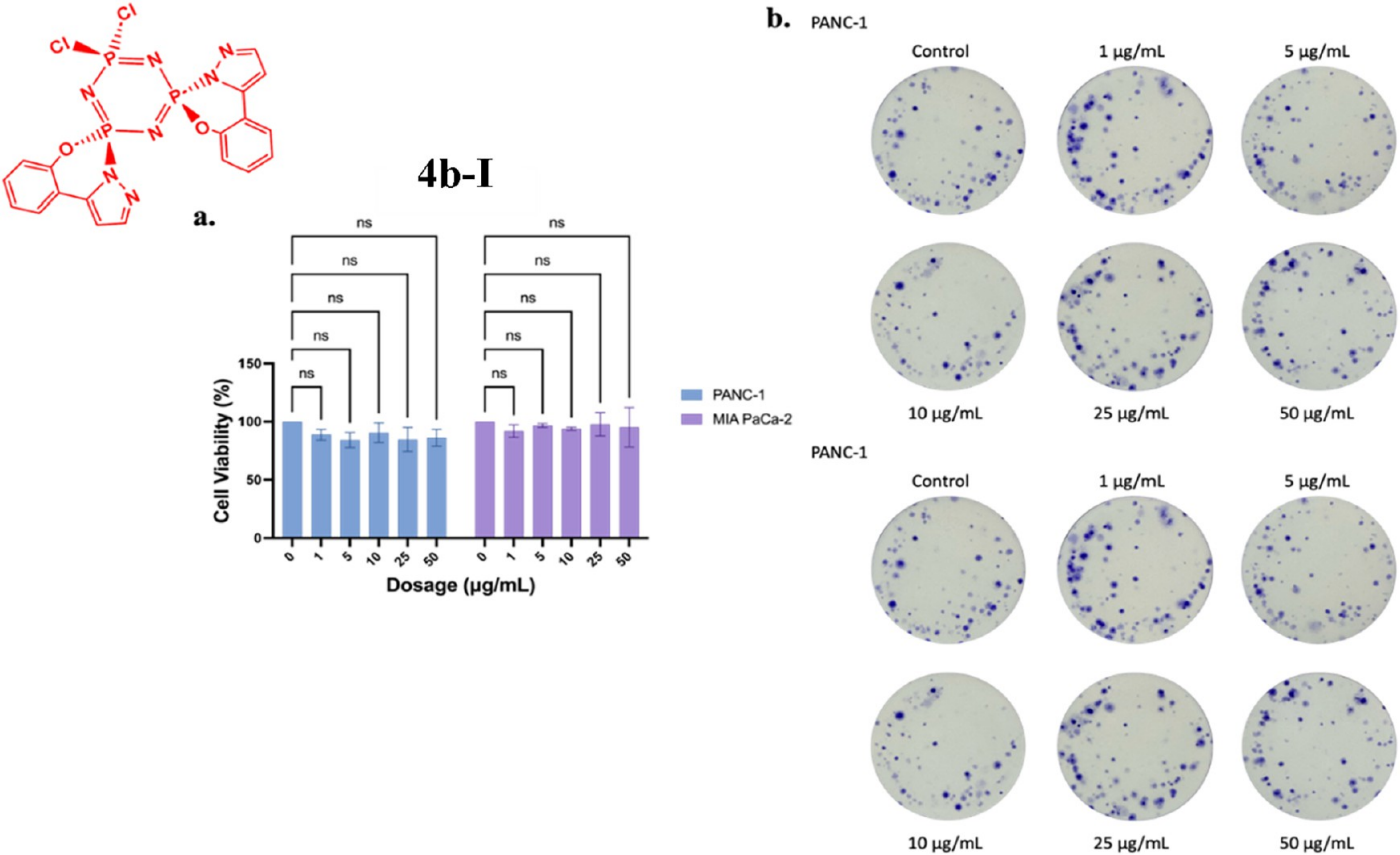
Effects of **4b-I** on (a) cell viability and (b) colony formation in PANC-1 and MIA-PaCa-2 cells following 24-h treatment. Data represents the MTT assay mean values ± standard deviation from four technical and three biological replicates. Two-way ANOVA determined statistical significance with Dunnett’s posthoc test (ns: *p* > 0.05).

Fluorescence imaging in both cell lines revealed no significant dose-dependent alterations. H_2_-DCFDA (ROS indicator), MitoTracker (mitochondrial mass), and DAPI (nuclear stain) showed no qualitative differences in reactive oxygen species production or mitochondrial activity across the tested concentrations of **4b-I** in both cell lines (Figures S12 and S13).

Treatment of PANC-1 and MIA PaCa-2 pancreatic cancer cells with increasing concentrations of **5b-I** (1, 5, 10, 25, and 50 μg/mL) did not significantly affect cell viability or colony-forming ability (Figure 17). Cell viability, as assessed by the MTT assay, remained consistently high (approximately 100%) across all tested concentrations in both cell lines, with no statistically significant differences between the treated and control groups. Similarly, the colony formation assay showed that the number and size of colonies formed were comparable between treated and untreated cells at all concentrations, indicating that **5b-I** does not impair the long-term proliferative capacity or clonogenic potential of PANC-1 and MIA-PaCa-2 cells. Overall, these results suggest that **5b-I** does not exert significant cytotoxic or antiproliferative effects on these pancreatic cancer cell lines at concentrations up to 50 μg/mL.

**Figure 17:**
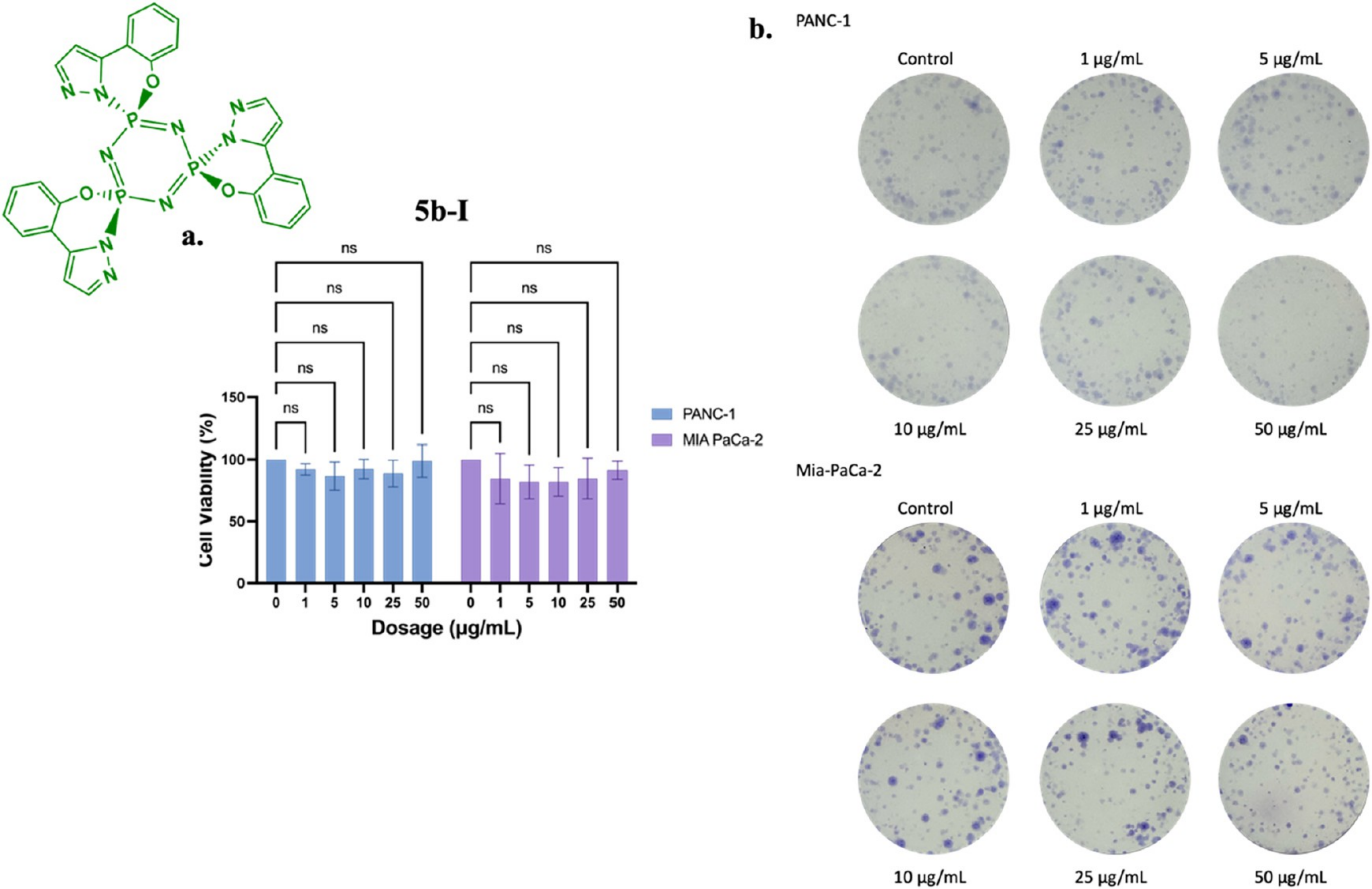
Effects of **5b-I** on (a) cell viability and (b) colony formation in PANC-1 and MIA-PaCa-2 cells following 24-h treatment. Data represent the MTT assay mean values ± standard deviation from four technical and three biological replicates. Two-way ANOVA determined statistical significance with Dunnett’s posthoc test (ns: *p* > 0.05).

Treatment of PANC-1 and MIA-PaCa-2 cells with increasing concentrations of **5b-I** (1, 5, 10, 25, and 50 μg/mL) for 24 h did not result in significant alterations in oxidative stress, mitochondrial integrity, or nuclear morphology compared to untreated controls (Figures S14 and S15). Across all tested concentrations, green fluorescence from DCFDA staining, indicative of intracellular reactive oxygen species, remained consistently low, suggesting that **5b-I** did not induce oxidative stress in either cell line. Similarly, red fluorescence from MitoTracker staining showed stable mitochondrial labeling, and blue fluorescence from DAPI indicated comparable numbers and distributions of nuclei in both treated and control groups. The merged images further confirmed these findings, demonstrating persistent colocalization of mitochondrial and nuclear signals without evidence of cytotoxicity or significant changes in cell health at any dose. Overall, these results indicate that **5b-I** does not provoke oxidative stress, mitochondrial dysfunction, or significant cell death in PANC-1 or MIA PaCa-2 cells under the experimental conditions used

The dose-dependent cytotoxic effects of the six synthesized compounds were evaluated in pancreatic cancer cell lines PANC-1 and MIA-PaCa-2 after 24 h of treatment using MTT and colony formation assays. The results were further supported by fluorescence staining (H2-DFDA, MitoTracker, DAPI, PI, DiOC6). In cellular experiments, the compound **3b** reduced colony formation in PANC-1 cells at 10 and 50 μg/mL and in MIA-PaCa-2 cells at 5, 10, and 50 μg/mL. The fluorescence dye results corroborate these findings.

Ebenezer et al. (2022) reported that pyrazole conjugates bearing para-chlorophenyl substituents display potent antiproliferative activity, with certain derivatives exhibiting IC_50_ values in the low micromolar range against HeLa cells, often rivalling or surpassing standard chemotherapy agents such as camptothecin; apoptosis induction and increased ROS were identified as part of their mechanism of action, explicitly attributed to halogen substitution on the aromatic ring.^44^ Furthermore, a study by Halim et al. synthesized a series of halogenated pyrazole derivatives and reported that those containing chlorine atoms had substantially higher cytotoxic activity against various cancer cell lines, including hepatocellular carcinoma, than their nonhalogenated analogs. The authors attributed this to enhanced cell membrane penetration and increased interaction with intracellular targets.^45^ These literature findings collectively validate the hypothesis that the strong cytotoxic effect of compound **3a** is likely due to its unique monospiro and chloro-substituted structure, which enhances both selectivity and potency against cancer cells through improved membrane permeability and cellular uptake, corroborating the results of the present study.

The biological results obtained for the pyrazole-cyclotriphosphazene derivatives in this study are consistent with the current literature, particularly regarding the significant impact of molecular architecture and substituent effects on both cytotoxic and antimicrobial activities. Specifically, the lack of substantial cytotoxicity for most multispiro derivatives (such as **4a-I**, **4b-I**, **5a-I**, and **5b-I**) and nonchlorinated analogs (**3b**, **4b-I**, **5b-I**) parallels findings by Beytur et al. (2022), who reported that complex, highly substituted cyclotriphosphazenes generally display diminished cellular uptake and bioactivity due to steric hindrance and reduced interaction with biological targets (IC_50_ > 50 μg/mL).^46^ Similarly, Palabıyık et al. (2021) found that macrocyclic phosphazenes with increased rigidity rarely exhibit strong cytotoxic effects, emphasizing that both substituent electronics and scaffold flexibility are essential for potent anticancer activity.^47^

### 3.5 Antimicrobial Effect

Antimicrobial activity was evaluated for all synthesized compounds except **5a-I** against Gram-negative (*E. coli*, *P. aeruginosa*) strains using disk diffusion and MIC assays, with ciprofloxacin (2 mg/mL) as a positive control; compound **5a-I** was not included in the antimicrobial assay because it was prioritized for cellular studies after showing no cytotoxic effect on PANC-1 and MIA-PaCa-2 cell lines (Figure S16). Only **4b-I** showed the inhibition zone in disk diffusion in *E. coli* (Figure S16); however, with Gram-positive *S. aureus*, no inhibition zone was observed for 5 tested samples (data not represented). MIC values determined by the broth microdilution assay showed measurable antibacterial activity for selected compounds against *E. coli, P. aeruginosa*, and *S. aureus* (Table S1). Compound **3a** exhibited MIC values of 0.75 ± 0.014 mg/mL against *E. coli* and 0.375 ± 0.002 mg/mL against *P. aeruginosa*, whereas **4a-I** showed MIC values of 0.375 ± 0.004 mg/mL against *E. coli* and 0.375 ± 0.006 mg/mL against *P. aeruginosa* (Table S1). Compound **4b-I** displayed MIC values of 0.75 ± 0.006 mg/mL against *E. coli* and 0.375 ± 0.001 mg/mL against *P. aeruginosa*, while no MIC was detected for **3b** and **5b-I** under the tested concentration range against either *E. coli* or *P. aeruginosa* (Table S1). Additionally, no MIC was detected against *S. aureus* for all tested compounds; therefore, MIC data for *S. aureus* were not presented.

On the antimicrobial studies such as Radini et al. (2018) and Okumuş et al. (2020) demonstrate that modifications on the pyrazole ring and overall molecular shape determine spectrum and potency-supporting the observation that only specific derivatives (such as **4b-I** against *E. coli)* showed moderate activity, while most others were ineffective due to bulky or deactivating groups.^48,49^ These literature comparisons confirm that the modest to absent biological activity of the majority of the synthesized compounds in this work is a direct outcome of their steric environment, electronic features, and the precise arrangement of their spiro/phosphazene motifs, reinforcing established principles in heterocycle-phosphazene drug design.

## 4 Conclusion

In this study, a series of novel inorganic–organic hybrid cyclotriphosphazene derivatives including the biologically active pyrazole scaffold were successfully synthesized and fully characterized using spectroscopic and crystallographic techniques. The use of different bases (NaH and Et_3_N) allowed the selective formation of mono-, di-, and trispiro derivatives, highlighting the influence of reaction conditions on product distribution. Notably, the biological evaluation of selected compound **3a** demonstrated potential activity against pancreatic cancer cell lines (PANC-1 and MIA PaCa-2), suggesting that this novel hybrid molecule could serve as a promising candidate for further anticancer drug development. Overall, the combination of the pyrazole pharmacophore with the cyclotriphosphazene scaffold provides a versatile platform for designing potent therapeutic agents against pancreatic cancer, while future studies will incorporate nonmalignant (healthy) pancreatic/epithelial cell lines to assess selectivity and better define the therapeutic window of the lead compounds.

## Supplementary Material



## Notes

Figure S1. ^31^P {H} NMR spectrumof compound **4a-I**; Figure S2. ^1^H NMR spectrum of compound **4a-I**; Figure S3. ^13^C NMR spectrum of compound **4a-I**; Figure S4. ^31^P {H} NMR spectrumof compound **5a-I**; Figure S5. ^1^H NMR spectrum of compound **5a-I**; Figure S6. ^13^C NMR spectrum of compound **5a-I**; Figure S7. The mass spectrum of compound **4a-I**; Figure S8. Fluorescence microscopy images of PANC-1 cells after 24-hour treatment with **4a-I**, staining of H_2_-DCFDA (green, ROS), MitoTracker (red, mitochondria), and DAPI (blue, nuclei), along with their merged image. Scale bar is 150 μm; Figure S9. Fluorescence microscopy images of MIA-PaCa-2 cells after 24-hour treatment with **4a-I**, staining of H_2_-DCFDA (green, ROS), MitoTracker (red, mitochondria), and DAPI (blue, nuclei), along with their merged image. Scale bar is 150 μm; Figure S10. Fluorescence microscopy images of PANC-1 cells after 24-hour treatment with **3b**, staining of H_2_-DCFDA (green, ROS), MitoTracker (red, mitochondria), and DAPI (blue, nuclei), along with their merged image. Scale bar is 150 μm; Figure S11. Fluorescence microscopy images of MIA-PaCa-2 cells after 24-hour treatment with 3b, staining of H_2_-DCFDA (green, ROS), MitoTracker (red, mitochondria), and DAPI (blue, nuclei), along with their merged image. Scale bar is 150 μm; Figure S12. Fluorescence microscopy images of PANC-1 cells after 24-hour treatment with **4b-I**, staining of H_2_-DCFDA (green, ROS), MitoTracker (red, mitochondria), and DAPI (blue, nuclei), along with their merged image. Scale bar is 150 μm; Figure S13. Fluorescence microscopy images of MIA-PaCa-2 cells after 24-hour treatment with **4b-I**, staining of H_2_-DCFDA (green, ROS), MitoTracker (red, mitochondria), and DAPI (blue, nuclei), along with their merged image. Scale bar is 150 μm; Figure S14. Fluorescence microscopy images of PANC-1 cells after 24-hour treatment with **5b-I**, staining of H_2_-DCFDA (green, ROS), MitoTracker (red, mitochondria), and DAPI (blue, nuclei), along with their merged image. Scale bar is 150 μm; Figure S15. Fluorescence microscopy images of MIA-PaCa-2 cells after 24-hour treatment with **5b-I**, staining of H_2_-DCFDA (green, ROS), MitoTracker (red, mitochondria), and DAPI (blue, nuclei), along with their merged image. Scale bar is 150 μm; Figure S16. Disk diffusion test results of synthesized compounds in *E. coli* and *P. aeruginosa* (red); Table S1. The antimicrobial effective concentrations of compounds in gram-negative bacteria after MIC analysis (PDF)
