# Supplementary material for: Pyrazole–Cyclotriphosphazene Hybrids: Synthesis, Structural Insights, and Cytotoxic Effects against Pancreatic Cancer Cells
Source: ACS Omega. 2026 Apr 8;11(15):22868–87. doi: 10.1021/acsomega.5c11955 (PMC13103825; doi:10.1021/acsomega.5c11955)
Supplement: Supplementary file 1 [file ao5c11955_si_004.pdf]

Pyrazole-Cyclotriphosphazene Hybrids: Synthesis, Structural Insights, and Cytotoxic Effects Against Pancreatic Cancer Cells

Ceylan Mutlu Balcı[a], Basak Dalbayrak[b], Esma Mutlu[a], Duygu Palabıyık[a], Elif Damla Arısan[b*]*

[a]Department of Chemistry, Faculty of Basic Sciences, Gebze Technical University, Gebze 41400 Kocaeli, Turkey

[b]Institute of Biotechnology, Gebze Technical University, Gebze 41400 Kocaeli, Turkey

Supporting Information

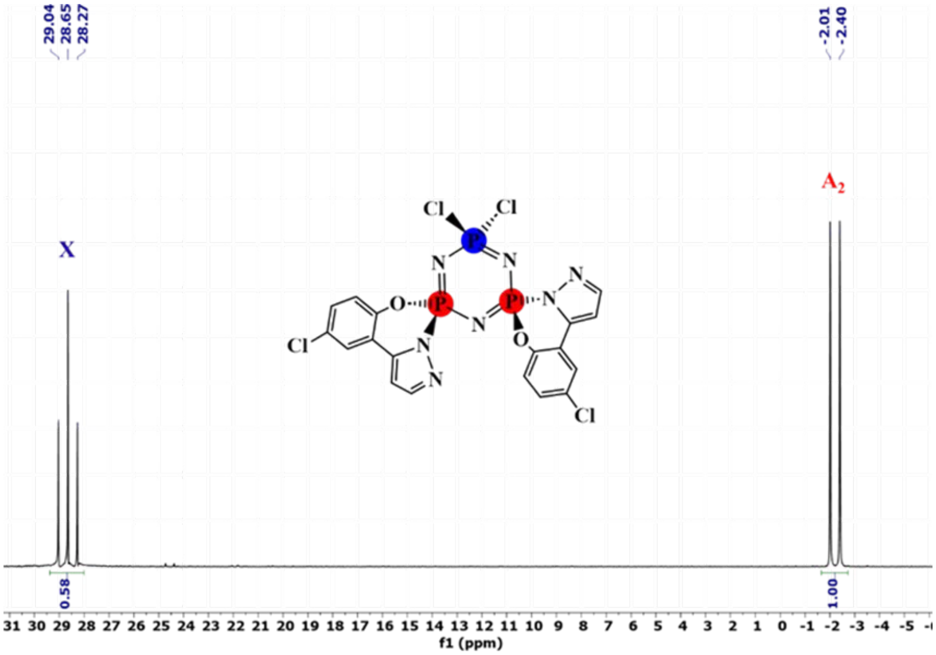

Figure S1. ^{31}P {H} NMR spectrum of compound **4a-I.**

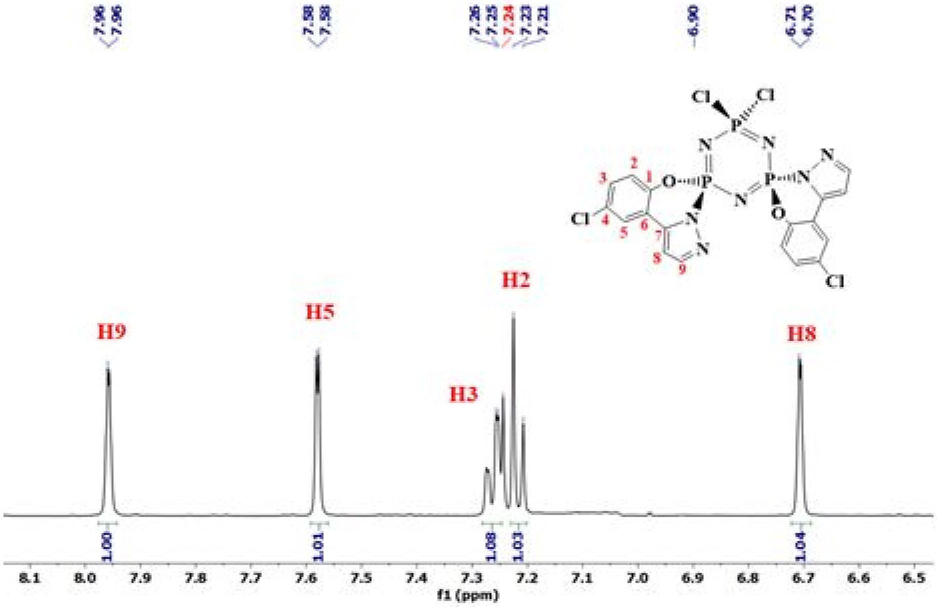

Figure S2. ^{1}H NMR spectrum of compound **4a-I.**

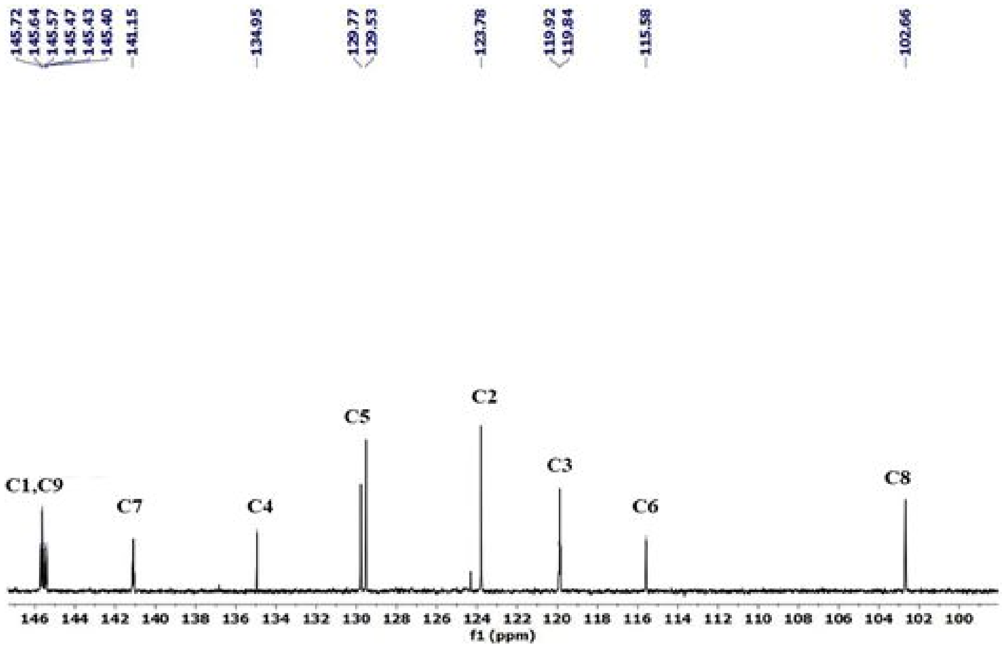

Figure S3. ^{13}C NMR spectrum of compound **4a-I.**

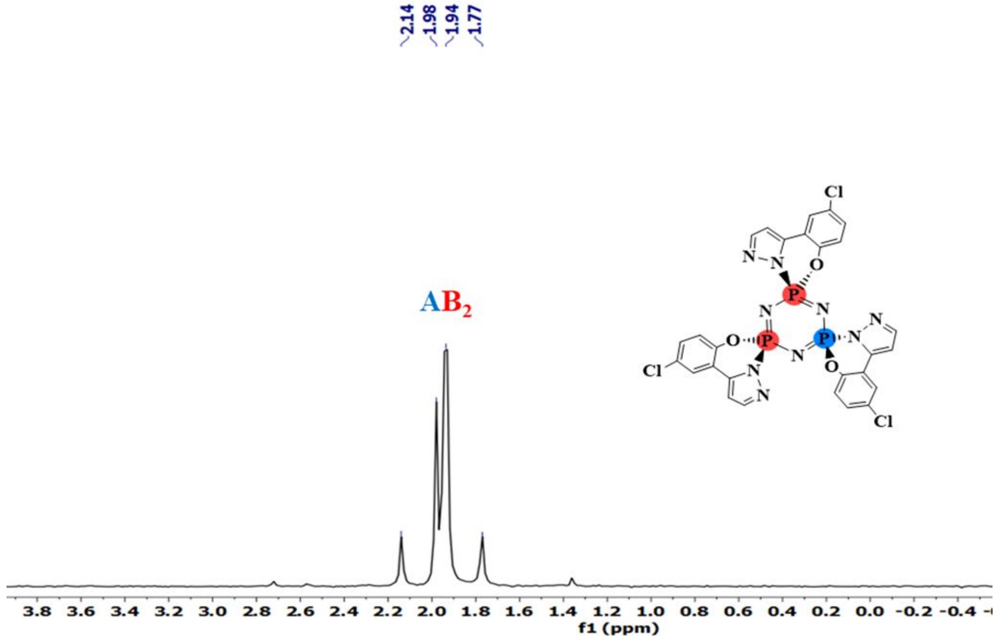

Figure S4. ^{31}P {H} NMR spectrum of compound **5a-I.**

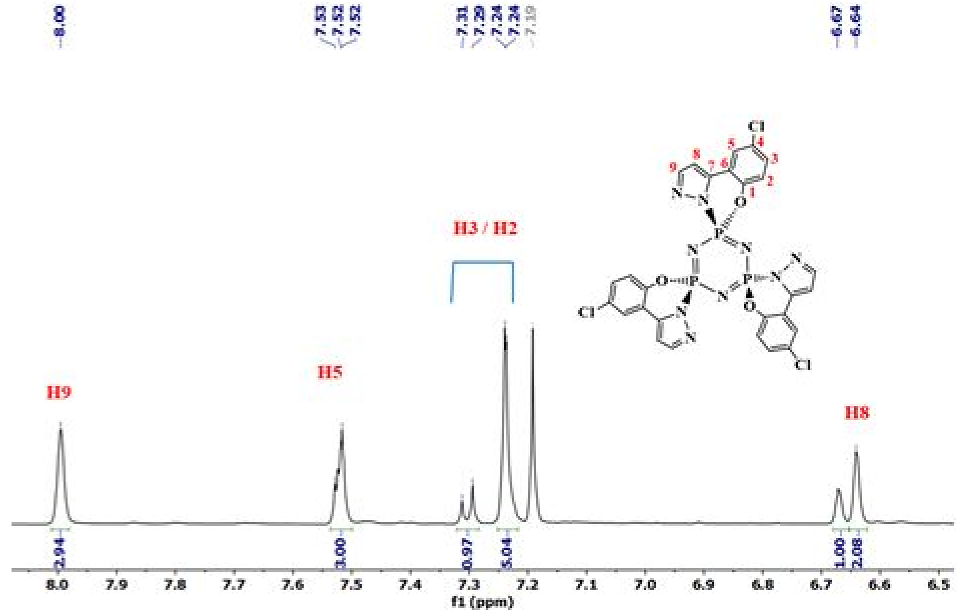

Figure S5. ^{1}H NMR spectrum of compound **5a-I.**

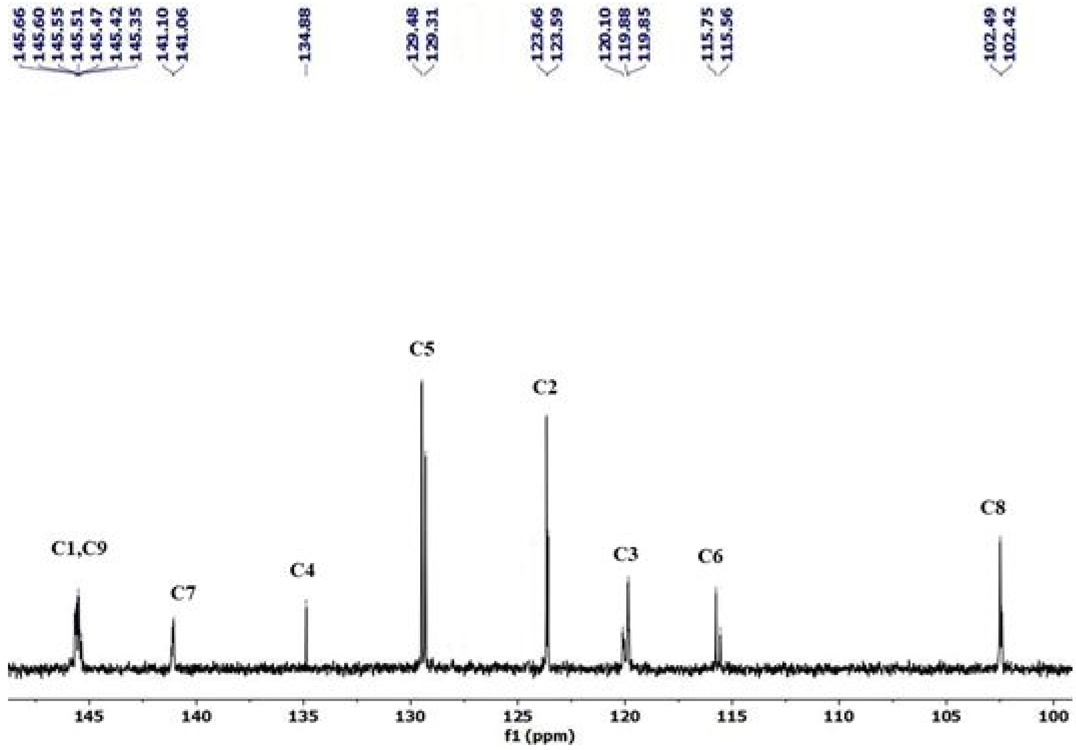

Figure S6. ^{13}C NMR spectrum of compound **5a-I.**

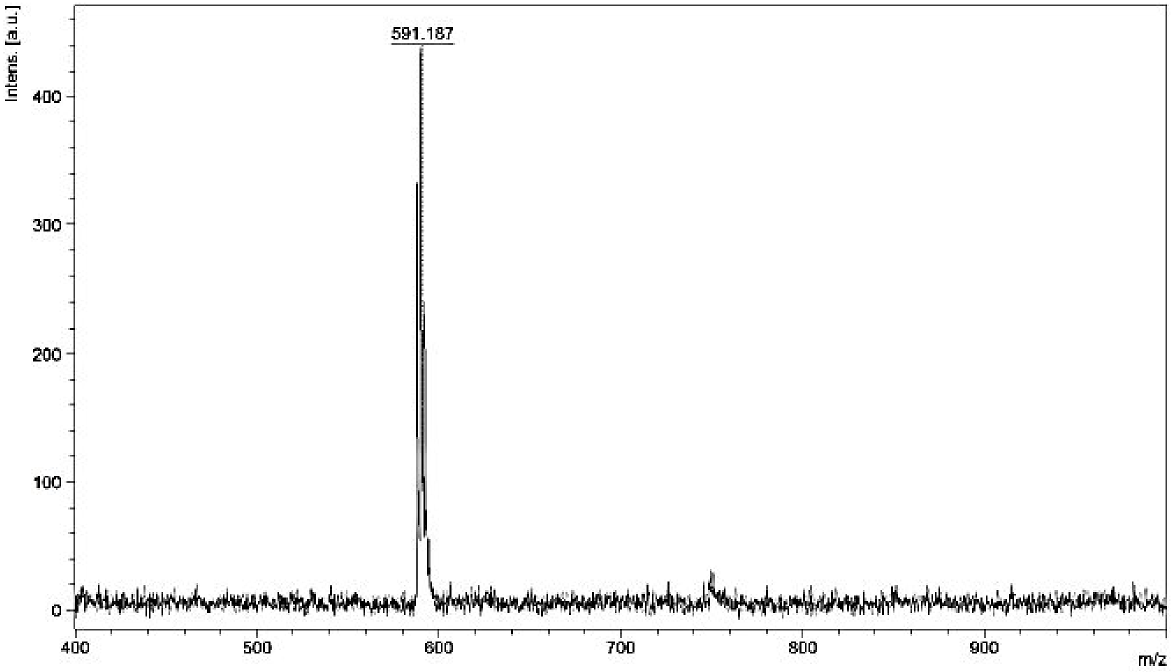

Figure S7. The mass spectrum of compound **4a-I**

Antimicrobial Effects

The antibacterial effects of the obtained compounds were first tested using the disk diffusion method with microorganisms (specifically, gram-positive *Pseudomonas aeruginosa* and gram-negative *Escherichia coli*). Sterilized paper discs (6 mm diameter filter paper) were impregnated with the samples in a DMSO-containing solution. 100 µL of bacterial inoculum ($8x10^8$ CFU/mL) was spread on the surface of Luria Broth (LB) Agar plates. For each compound, 10 µL of solution was loaded onto disks, which were then placed on the bacteria spread plates. Disks saturated with 2 mg/mL Ciprofloxacin as a positive control and DMSO-soaked disks as solvent control were also used for inhibition zone analysis. LB Agar plates streaked with bacteria and plated with samples

were incubated overnight at 37°C. The inhibition zones around the disks were subtracted from those caused by DMSO, and the antimicrobial properties of the compounds were determined.

Ericsson and Sherris (1971) developed the microdilution method to determine the minimum inhibitory concentration (MIC) of the compounds. The compounds were dissolved in DMSO at an initial concentration of 3 mg/mL, diluted 8 times using a serial dilution technique with LB Broth, and incubated under the same conditions as disk diffusion, with constant bacteria. LB Broth without bacteria was used as a negative control, and solutions containing equal concentrations of bacteria Lin B were used as a positive control. MIC values were recorded as the lowest concentration of antibacterial agents that inhibited bacterial growth and were read at 600 nm using a spectrophotometer (Shimadzu).

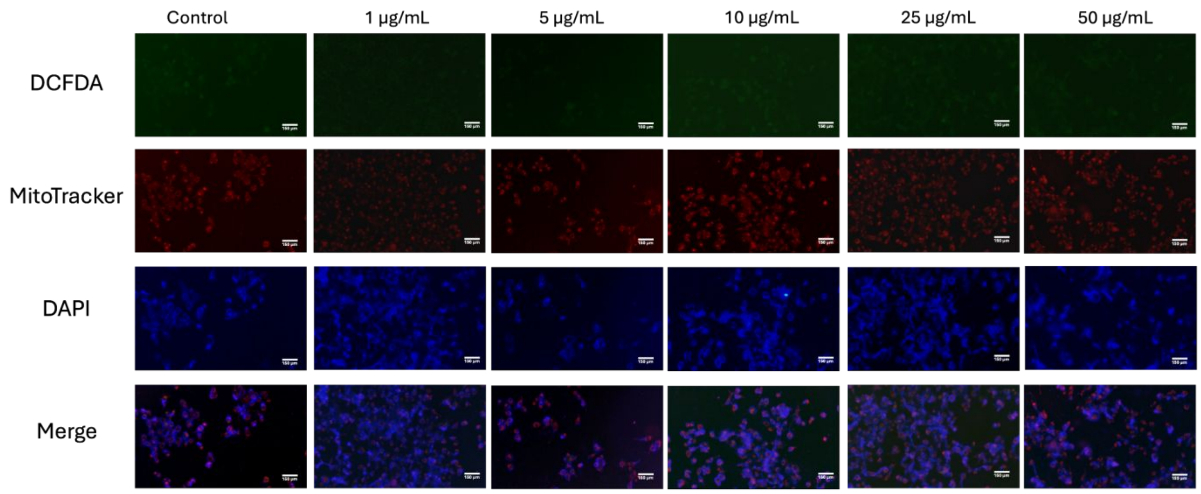

Figure S8. Fluorescence microscopy images of PANC-1 cells after 24-hour treatment with **4a-I**, staining of H_2-DCFDA (green, ROS), MitoTracker (red, mitochondria), and DAPI (blue, nuclei), along with their merged image. Scale bar is 150 µm.

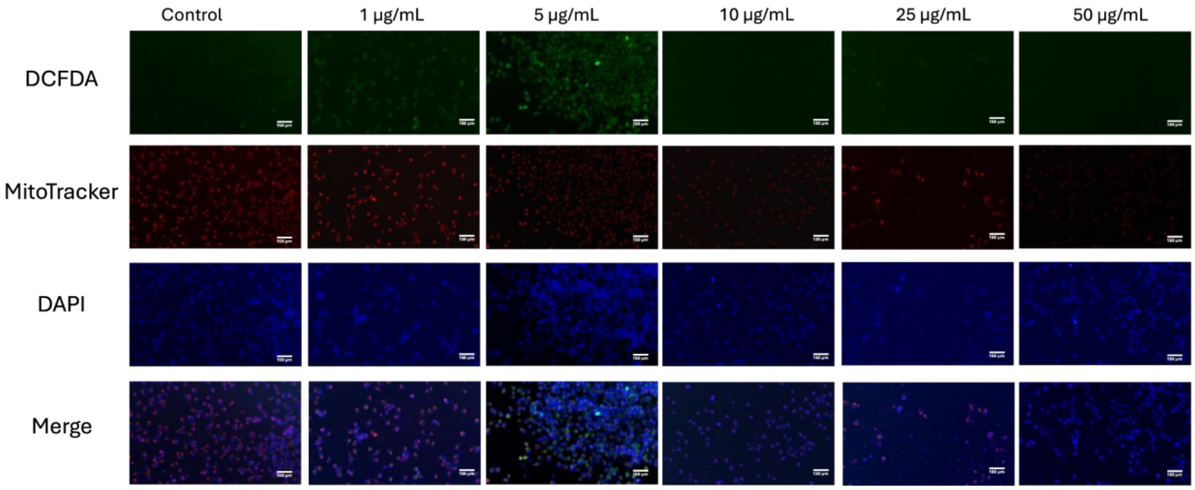

Figure S9. Fluorescence microscopy images of MIA-PaCa-2 cells after 24-hour treatment with **4a-I**, staining of H_2-DCFDA (green, ROS), MitoTracker (red, mitochondria), and DAPI (blue, nuclei), along with their merged image. Scale bar is 150 μm.

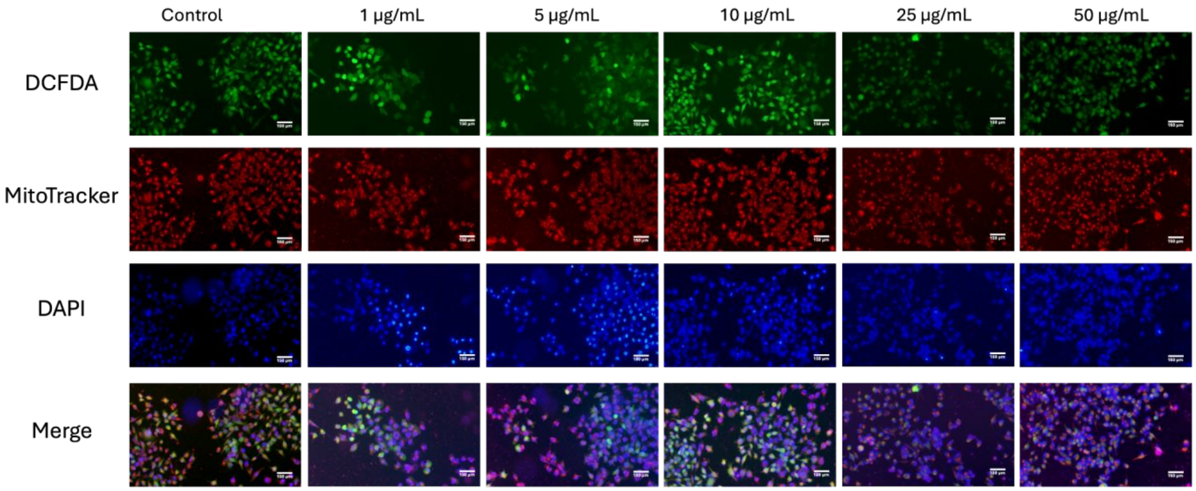

Figure S10. Fluorescence microscopy images of PANC-1 cells after 24-hour treatment with **3b**, staining of H_2-DCFDA (green, ROS), MitoTracker (red, mitochondria), and DAPI (blue, nuclei), along with their merged image. Scale bar is 150 µm.

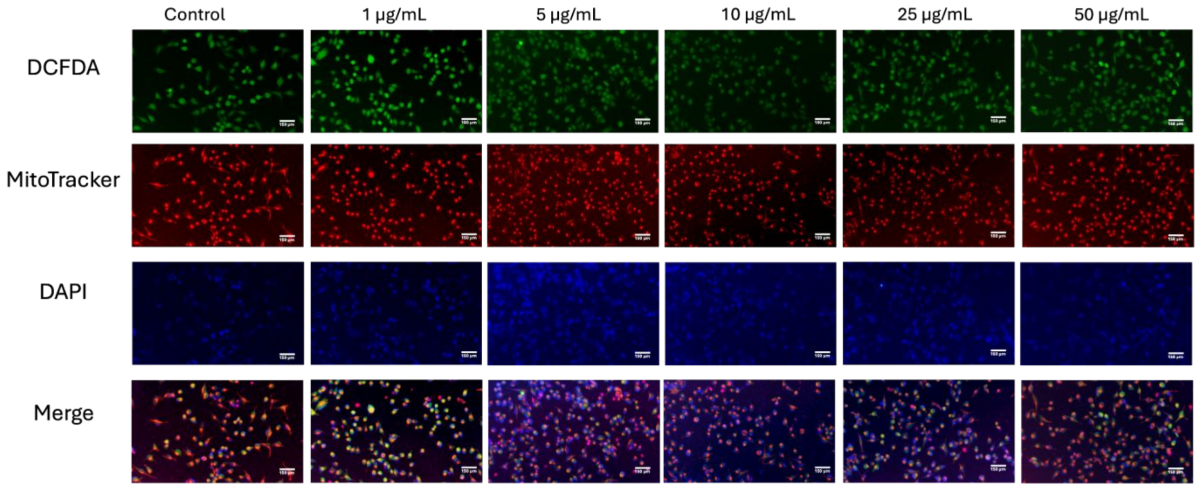

Figure S11. Fluorescence microscopy images of MIA-PaCa-2 cells after 24-hour treatment with **3b**, staining of H_2-DCFDA (green, ROS), MitoTracker (red, mitochondria), and DAPI (blue, nuclei), along with their merged image. Scale bar is 150 µm.

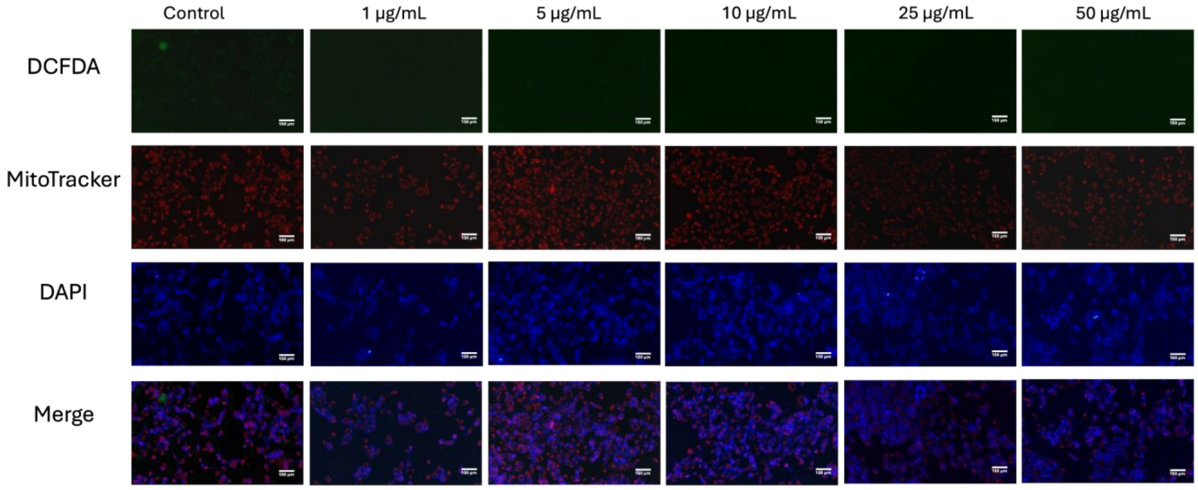

Figure S12. Fluorescence microscopy images of PANC-1 cells after 24-hour treatment with **4b-I**, staining of H_2-DCFDA (green, ROS), MitoTracker (red, mitochondria), and DAPI (blue, nuclei), along with their merged image. Scale bar is 150 µm.

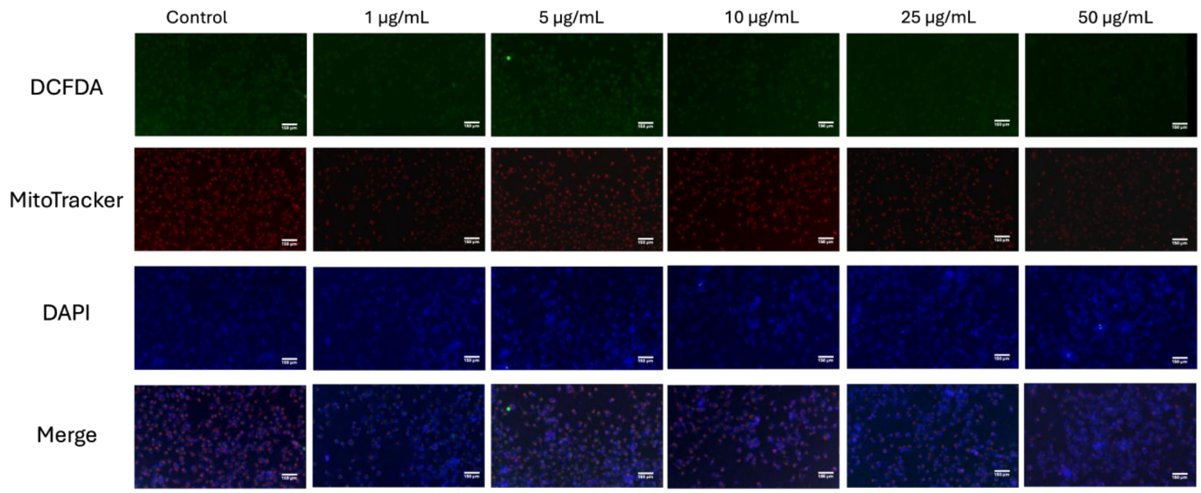

Figure S13. Fluorescence microscopy images of MIA-PaCa-2 cells after 24-hour treatment with **4b-I**, staining of H_2-DCFDA (green, ROS), MitoTracker (red, mitochondria), and DAPI (blue, nuclei), along with their merged image. Scale bar is 150 µm.

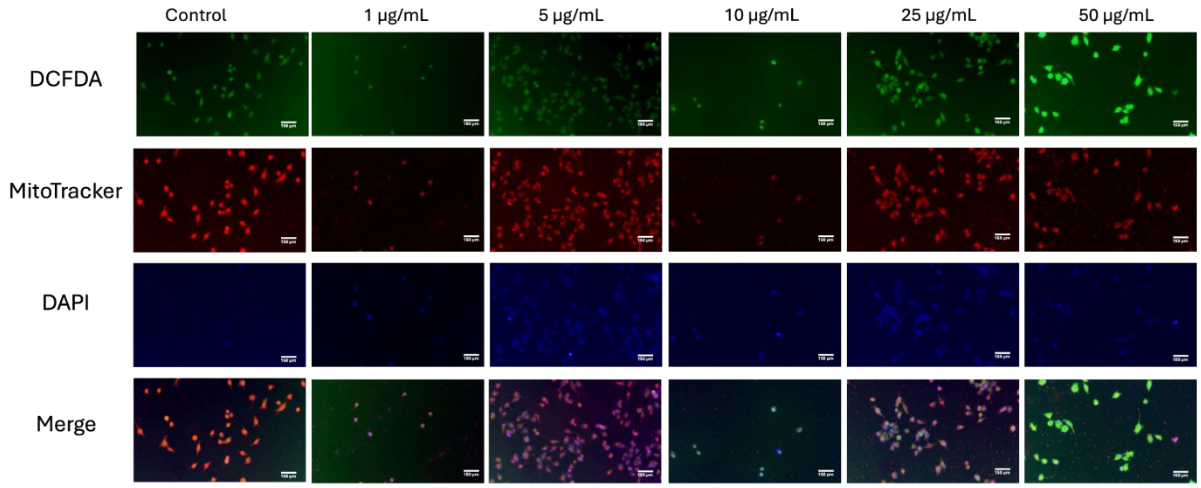

Figure S14. Fluorescence microscopy images of PANC-1 cells after 24-hour treatment with **5b-I**, staining of H_2-DCFDA (green, ROS), MitoTracker (red, mitochondria), and DAPI (blue, nuclei), along with their merged image. Scale bar is 150 µm.

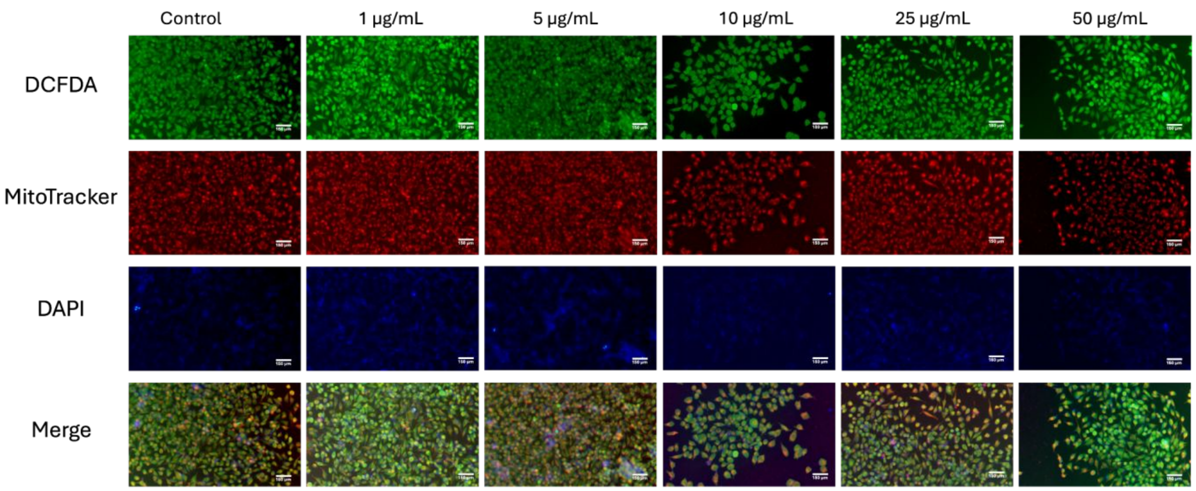

Figure S15. Fluorescence microscopy images of MIA-PaCa-2 cells after 24-hour treatment with **5b-I**, staining of H_2-DCFDA (green, ROS), MitoTracker (red, mitochondria), and DAPI (blue, nuclei), along with their merged image. Scale bar is 150 µm.

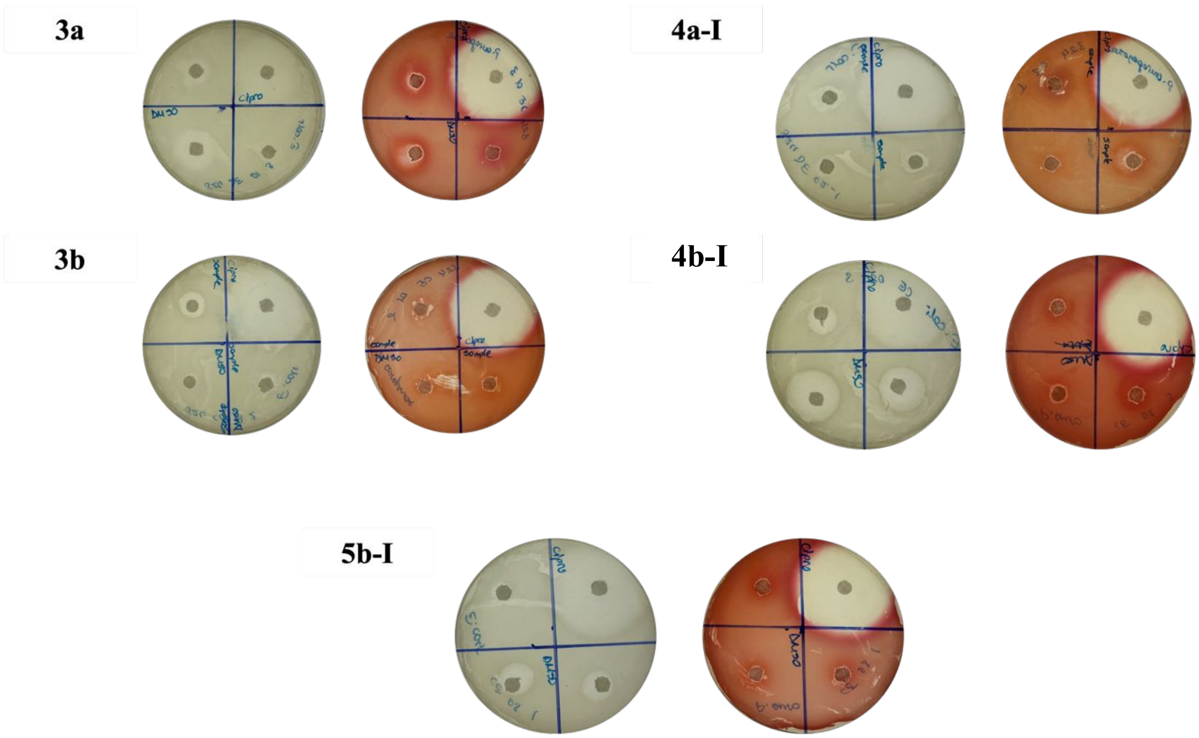

Figure S16. Disk diffusion test results of synthesized compounds in *E. coli* and *P. aeruginosa (red)*

Table S1. The antimicrobial effective concentrations of compounds in gram-negative bacteria after MIC analysis.

Minimum inhibition concentration (mg/ml)	*E.coli*	*P.aeruginosa*
**3a**	0.75 ± 0.014	0.375 ± 0.002
**4a-I**	0.375 ± 0.004	0.375 ± 0.006
**3b**	-	-
**4b-I**	0.75 ± 0.006	0.375 ± 0.001
**5b-I**	-	-
